# Necdin: A purposive integrator of molecular interaction networks for mammalian neuron vitality

**DOI:** 10.1111/gtc.12884

**Published:** 2021-08-02

**Authors:** Kazuaki Yoshikawa

**Affiliations:** ^1^ Professor Emeritus, Osaka University Suita Japan

**Keywords:** DNA damage response, genomic imprinting, MAGE family, mammalian brain, necdin, neuronal development, neuronal vitality, neuroprotection, Prader‐Willi syndrome, protein‐protein interaction network

## Abstract

Necdin was originally found in 1991 as a hypothetical protein encoded by a neural differentiation‐specific gene transcript in murine embryonal carcinoma cells. Virtually all postmitotic neurons and their precursor cells express the necdin gene (Ndn) during neuronal development. Necdin mRNA is expressed only from the paternal allele through genomic imprinting, a placental mammal‐specific epigenetic mechanism. Necdin and its homologous MAGE (melanoma antigen) family, which have evolved presumedly from a subcomplex component of the SMC5/6 complex, are expressed exclusively in placental mammals. Paternal Ndn‐mutated mice totally lack necdin expression and exhibit various types of neuronal abnormalities throughout the nervous system. Ndn‐null neurons are vulnerable to detrimental stresses such as DNA damage. Necdin also suppresses both proliferation and apoptosis of neural stem/progenitor cells. Functional analyses using Ndn‐manipulated cells reveal that necdin consistently exerts antimitotic, anti‐apoptotic and prosurvival effects. Necdin interacts directly with a number of regulatory proteins including E2F1, p53, neurotrophin receptors, Sirt1 and PGC‐1α, which serve as major hubs of protein–protein interaction networks for mitosis, apoptosis, differentiation, neuroprotection and energy homeostasis. This review focuses on necdin as a pleiotropic protein that integrates molecular interaction networks to promote neuronal vitality in modern placental mammals.

## Graphical abstract

Necdin interacts with major regulatory proteins such as E2F1, p53, neurotrophin receptors (TrkA, p75NTR), Sirt1 and PGC‐1α, which serve as highly connected hubs of protein–protein interaction networks for mitosis, apoptosis, differentiation, neuroprotection and energy homeostasis. By integrating these networks, necdin promotes the vitality of mammalian neurons.

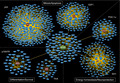

**Abbreviations**
aa: amino acid(s)
EC: embryonal carcinoma
MAGE: melanoma antigen
NSPC: neural stem/progenitor cell
PWS: Prader–Willi syndrome

## 1 INTRODUCTION

Neurons are extremely specialized cells that form complex networks for electrical signal transduction and processing. These cells differentiate from highly proliferative precursor cells and become permanently incapable of cell division during early development. Through this process known as terminal differentiation, most, if not all, neurons in mammals enter the postmitotic state during the embryonic period. Therefore, most of human neurons, for example, have potential abilities to survive over a century, imaginably beyond the lifespan of their host. Since neurons suffer from many detrimental stresses throughout the lifetime, deficiencies in neuroprotective mechanisms may cause neurodevelopmental defects and neurodegenerative diseases such as Alzheimer's disease and Parkinson's disease. Accordingly, neurons must possess intrinsic mechanisms that maintain their vitality during the long‐lasting postmitotic period.

Cultured cells that differentiate in vitro into postmitotic neurons provide useful tools for molecular analyses of neurodevelopmental and neuropathological events. Embryonal carcinoma (EC) cell lines such as murine P19 cells (McBurney et al., 1982) and human NTERA‐2 cells (Andrews, 1984) are pluripotent stem cells that differentiate efficiently into postmitotic neurons. P19 EC cells treated with retinoic acid differentiate into postmitotic neurons and then into glial cells (McBurney, 1993). The neural differentiation process of EC cells resembles that of mammalian brain cells in vivo and in vitro (Temple, 2001; Yoshikawa, 2000). Furthermore, postmitotic neurons derived from these EC cells are vulnerable to over‐expression of APP (amyloid precursor protein) (Uetsuki et al., 1999; Yoshikawa et al., 1992) and E2F1 (Azuma‐Hara et al., 1999), both of which are implicated in neuropathological events. Consequently, these EC cells are suitable in vitro systems for analyzing the molecular mechanisms underlying neurodevelopmental and neuropathological processes (McBurney, 1993; Yoshikawa, 2000).

A neuronal differentiation‐specific transcript was isolated from a cDNA library of retinoic acid‐treated P19 EC cells (Maruyama et al., 1991). The transcript encoded a hypothetical protein comprising 325 amino acid (aa) residues that showed no sequence homology to known proteins. This protein was thus designated as necdin for neurally differentiated EC cell‐derived protein. The research on necdin has been greatly facilitated by two key discoveries. First, necdin shared a homology domain with MAGE (melanoma antigen) family proteins, which were expressed exclusively in placental mammals (De Backer et al., 1995; Chomez et al., 2001; De Plaen et al., 1994). Therefore, necdin presumedly emerged through MAGE gene diversification during the evolution of placental mammals. Second, human necdin gene (NDN) was located at the chromosomal region involved in the pathogenesis of Prader–Willi syndrome (PWS), a classic genomic imprinting‐associated neurodevelopmental disorder (Jay et al., 1997; MacDonald & Wevrick, 1997; Sutcliffe et al., 1997). Genomic imprinting is a typical DNA methylation‐dependent epigenetic control of gene expression seen only in placental mammals. Necdin was normally expressed only from the paternal allele, which was mutated in PWS. These findings suggested certain roles of necdin in neuronal development of placental mammals.

Physiological roles of necdin in neuronal development have been explored using necdin‐deficient mice that lack the paternal necdin gene. These mice show morphological and biochemical abnormalities throughout the nervous system. Functional analyses using necdin‐deficient cells have shown that necdin efficiently suppresses the mitosis and apoptosis of neuronal precursors, whereas it promotes the differentiation and survival of postmitotic neurons. Necdin‐deficient neurons are highly susceptible to various DNA damage‐inducing insults. Molecular and cellular analyses of these neuronal phenotypes have revealed that necdin promotes neuronal vitality by interacting with many regulatory proteins. This review summarizes the progress of multidisciplinary research on necdin in the last thirty years and discusses the current understanding of necdin including its implications of developmental neurobiology, neuropathology and mammalian brain evolution.

## 2 GENE EXPRESSION

### 2.1 cDNA cloning

**FIGURE 1:**
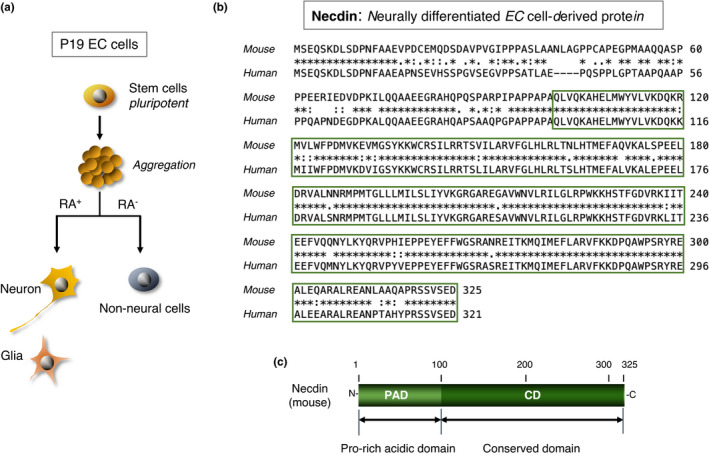
Necdin mRNA is expressed in neurally differentiated embryonal carcinoma cells. (a) Diagram of neural differentiation of P19 embryonal carcinoma (EC) cells. P19 EC cells are aggregated and treated with (RA^+^) or without (RA^−^) of retinoic acid. Clones of RA^+^ cell‐specific cDNAs were screened for neuron‐specific expression. For experimental details, see Maruyama et al. (1991). (b) Alignment of mouse and human necdin proteins. Necdin (mouse) is encoded by a cloned RA^+^ cell‐specific cDNA. Human necdin DNA (human) was cloned from a human genomic DNA library using mouse necdin cDNA. Sequences highly conserved between mouse and human are boxed. Sequence data from Maruyama et al. (1991) and Nakada et al. (1998). (c) Domain structure of mouse necdin. Necdin (mouse) consists of an N‐terminal 100 aa domain rich in proline/acidic aa (PAD) and a highly conserved 225 aa domain (CD)

Necdin cDNA was isolated from a neural differentiation‐specific cDNA library of retinoic acid‐treated P19 EC cells that differentiate into postmitotic neurons and glial cells (Maruyama et al., 1991) (Figure 1a). Necdin mRNA levels increase rapidly after retinoic acid treatment, reach a plateau and decline thereafter. This expression pattern is consistent with the appearance and disappearance of postmitotic neurons in retinoic acid‐treated P19 cell cultures (Aizawa et al., 1991; Yoshikawa et al., 1990). Sequence alignment of mouse necdin (325 aa residues) and human necdin (321 aa residues) reveals that necdin has a large conserved domain of ~200 aa residues at the carboxyl (C)‐terminus (Nakada et al., 1998) (Figure 1b). A less conserved 100 aa residue domain at the amino (N)‐terminus is rich in proline and acidic aa (calculated pI = 4.0 for humans; 3.9 for mouse: the number of proline residues = 21 for both) (Figure 1c). The N‐terminal proline‐rich acidic region and the conserved C‐terminal region show 62% and 91% aa identity, respectively, between humans and mouse. The high conservation of the C‐terminal region suggests a strong functional constraint of this region.

### 2.2 Postmitotic neurons

**FIGURE 2:**
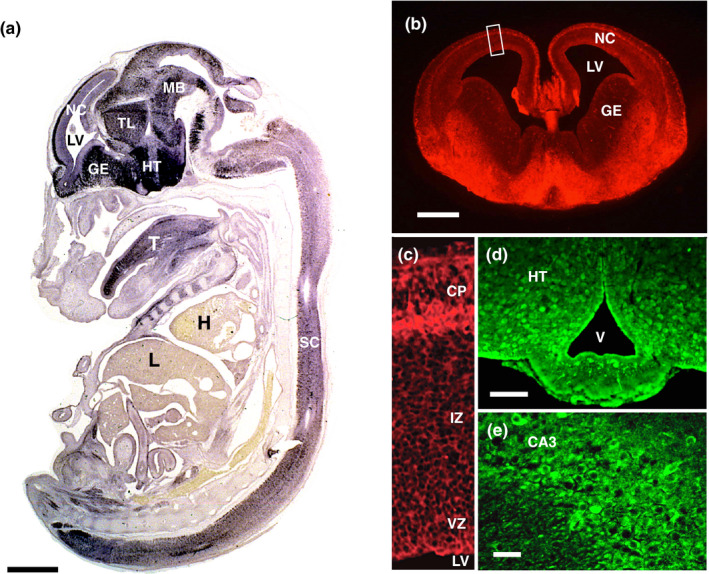
Necdin is strongly expressed in postmitotic neurons. (a) In situ hybridization histochemistry. Necdin mRNA expression in the mouse whole body (sagittal section) at embryonic day 14.5 (E14.5) was analyzed by in situ hybridization with digoxigenin‐labeled mouse necdin cRNA probe. For experimental details, see Uetsuki et al. (1996) (Takagi, K. & Yoshikawa, K., unpublished data). (b‐e) Immunohistochemistry. Frozen sections of mouse forebrain (b), neocortex (c), hypothalamus (d) and hippocampus (e) at E14.5 (b, c) and postnatal day 30 (d, e) were immunostained with anti‐necdin antibody NC243 (Niinobe et al., 2000). Boxed area (b) is enlarged (c). Abbreviations (a‐e): NC, neocortex; LV, lateral ventricle; GE, ganglionic eminence; TL, thalamus; HT, hypothalamus; MB, midbrain; SC, spinal cord; T, tongue; H, heart; L, liver; CP, cortical plate; IZ, intermediate zone; VZ, ventricular zone; V, ventricle; and CA3, Cornu ammonis area 3. Scale bars: 1 mm (a), 500 μm (b) and 50 μm (d, e) (Fujiwara, K., Fujimoto, I. & Yoshikawa, K., unpublished data)

Necdin mRNA is expressed in virtually all postmitotic neurons throughout the mouse nervous system (Aizawa et al., 1992; Andrieu et al., 2003; Uetsuki et al., 1996). In embryonic mouse brain in vivo, necdin mRNA is abundantly expressed in the hypothalamus, ganglionic eminences, cortical plate, thalamus and midbrain (Figure 2a). Necdin mRNA is also expressed in the spinal cord and skeletal muscle (tongue), whereas necdin mRNA levels are very low in the heart and liver. In adult mouse brain in vivo, necdin mRNA is highly expressed in phylogenetically old brain structures such as the hypothalamus, amygdaloid nuclei, piriform cortex and hippocampus (Uetsuki et al., 1996). In the neocortex, early‐born neurons located in the deep layers express higher levels of necdin mRNA than late‐born neurons in the surface layers. Moreover, necdin mRNA is expressed at high levels in spinal cord motor neurons, retinal ganglion cells and sympathetic postganglionic neurons (Yoshikawa, 2000). During early embryonic period of mouse development, necdin mRNA is expressed in non‐neural tissues such as the somites, limb buds and first branchial arches (Uetsuki et al., 1996; Yoshikawa, 2000). In contrast to the brain‐predominant expression of necdin mRNA in mice (Maruyama et al., 1991), non‐neural organs such as the heart, skeletal muscle and placenta express relatively high levels of necdin mRNA in humans (Jay et al., 1997; MacDonald & Wevrick, 1997).

The distribution pattern of the necdin protein in the brain is similar to that of its mRNA (Figure 2b‐e). Although neuronal nuclei are strongly immunostained with antipeptide antibodies used in the initial studies (Aizawa et al., 1992; Maruyama et al., 1991), immunocytochemistry using a more specific antibody raised against the C‐terminal 243 aa region of necdin (NC243), which recognizes a single necdin‐immunoreactive band at ~43 kDa in Western blots, reveals that necdin is mainly distributed in the cytosol of differentiated neurons (Niinobe et al., 2000). On the other hand, necdin localizes to both the nucleus and the cytoplasm, to varying extents, of cortical neurons in vivo in mouse embryos (Hasegawa & Yoshikawa, 2008; Kuwajima et al., 2006). Necdin is associated with the nuclear matrix (Taniura et al., 2005; Taniura & Yoshikawa, 2002) and transmembrane proteins (Kuwako et al., 2004, 2005). Therefore, necdin may localize to various subcellular compartments in a cell context‐dependent manner.

### 2.3 Stem/progenitor cells

**FIGURE 3:**
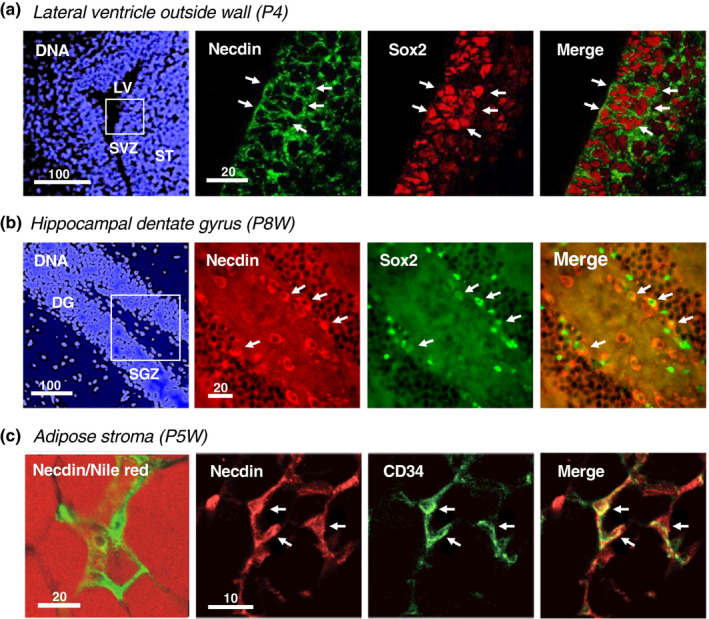
Necdin is expressed in postnatal stem/progenitor cells. (a, b**)** The lateral ventricle subventricular zone (SVZ) at postnatal day 4 (P4) (a) and the hippocampal dentate gyrus (DG) at postnatal week 8 (P8W) (b). Neural stem/progenitor cells were double‐immunostained for necdin and Sox2. Chromosomal DNA was stained with Hoechst33342 (DNA). Boxed areas (a, b) for immunohistochemistry. Abbreviations: SVZ, subventricular zone; ST, striatum; LV, lateral ventricle; SGZ, subgranular zone; and DG, dentate gyrus (Hashinaga, H. Fujimoto, I. & Yoshikawa, K., unpublished data). (c) White adipose tissues at P5W. Mesenchymal stromal cells were double‐immunostained for necdin and CD34. Adipocytes were stained with Nile red (red). Arrows (a‐c) point to double‐immunopositive cells for necdin and the stem cell marker Sox2 (a, b) or CD34 (c). Image data from Fujiwara et al. (2012). Numbers above scale bars in μm (a‐c)

Necdin is expressed in neural stem/progenitor cells (NSPCs), a mixed population of neural stem cells and neuronal progenitor cells, in the embryonic brain (Huang et al., 2013; Minamide et al., 2014). Additionally, necdin is present in postnatal NSPCs expressing the neural stem cell marker Sox2 at the subventricular zone (SVZ) near the lateral ventricle (Figure 3a) and at the subgranular zone (SGZ) of hippocampal dentate gyrus (Figure 3b). In these regions, neurogenesis continues until adulthood to replace old neurons (Zhao et al., 2008). Necdin is also present in melanocytes (Hoek et al., 2004), mesenchymal stromal cells (Fujiwara et al., 2012) (Figure 3c), human fibroblasts (Jay et al., 1997; MacDonald & Wevrick, 1997) and mouse embryonic fibroblasts (Bush & Wevrick, 2012). Furthermore, necdin is expressed in non‐neural stem cells/progenitor cells such as vessel‐derived stem cells (mesoangioblasts) (Brunelli et al., 2004), brown preadipocytes (Tseng et al., 2005), white preadipocytes (Fujiwara et al., 2012), hematopoietic stem cells (Kubota et al., 2009; Liu, Elf, et al., 2009), skeletal muscle satellite cell‐derived myoblasts (Deponti et al., 2007) and hepatic stellate cells (Zhu et al., 2010). In contrast, necdin expression is hardly detectable in most cell lines of neuroectodermal origin such as neuroblastoma, pheochromocytoma, glioma and ependymoma (Aizawa et al., 1992, 2011). Collectively, necdin may be expressed in neural and non‐neural stem/progenitor cells that differentiate into terminally differentiated cells.

### 2.4 Transcriptional regulation

The necdin gene (official gene symbol; Ndn for mouse or NDN for humans) lacks canonical TATA and CAAT boxes in their upstream promoter regions (Nakada et al., 1998; Uetsuki et al., 1996). The 5' flanking sequence (844 base pairs) of mouse Ndn directs expression in P19 cell‐derived postmitotic neurons and contains the proximal 80 base pair region that functions as a neuron‐restrictive core promoter (Uetsuki et al., 1996). Interestingly, the 5' flanking 844 base pair region directs expression in the brain and spinal cord of zebrafish in vivo (Kuo et al., 1995), indicating that neuronal expression of Ndn is mediated by its cis‐acting 5' flanking sequence.

**FIGURE 4:**
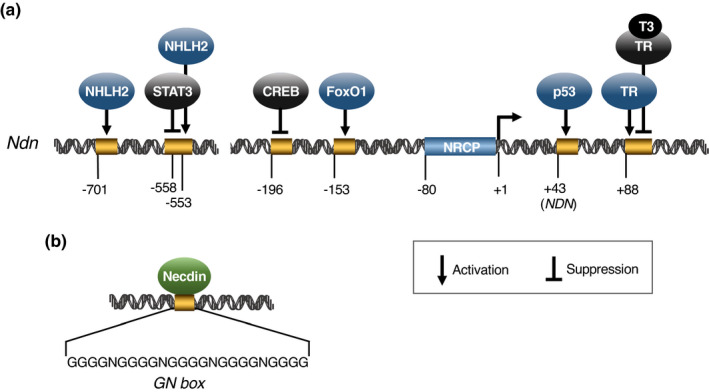
Necdin gene 5' flanking region contains binding sites of major transcription factors. (a) Transcription factors and their binding sites in mouse Ndn. Putative binding sites of transcription factors on Ndn 5' flanking region were experimentally determined: NHLH2 (Kruger et al., 2004), STAT3 (Haviland et al., 2011), CREB (Cypess et al., 2011), FoxO1 (Cypess et al., 2011), p53 (human NDN) (Liu, Elf, et al., 2009), TR (thyroid hormone receptor) (T3/TR for ligand‐bound TR) (Nygard et al., 2006) and NRCP (neuron‐restrictive core promoter) (Uetsuki et al.,^1996^). Transcription initiation site = +1. Nucleotide positions are from Uetsuki et al. (1996). Not drawn to scale. (b) Necdin‐binding GN box. Necdin‐binding sequences were selected by PCR‐based amplification of random‐sequence oligonucleotides bound to recombinant necdin. A typical GN box comprises multiple G clusters (~4Gs) and intervening mono‐ or di‐nucleotides of A, T and C (N). For experimental details, see Matsumoto et al. (2001)

Necdin expression is transcriptionally regulated by major transcription factors that bind directly to specific motifs in its 5' flanking sequence (Figure 4a). NHLH (nescient helix‐loop‐helix, also known as NSCL or HEN), a neural cell‐specific basic helix‐loop‐helix transcription factor (Begley et al., 1992), comprises two closely related genes (NHLH1 and NHLH2). NHLH2 transactivates Ndn by interacting with E‐boxes (NHLH‐binding motifs) located at −701 and −553 in the Ndn 5’ flanking region (Kruger et al., 2004). STAT3 (signal transducer and activator of transcription 3), a transcription factor mediating cytokine and growth factor signals, down‐regulates Ndn expression by interacting with a motif at −558 of the Ndn promoter (Haviland et al., 2011). Constitutively active CREB (cyclic AMP response element‐binding protein) represses Ndn expression via a putative binding motif at −196, whereas constitutively active FoxO1 (forkhead box protein O1) activates Ndn expression via a motif at −153 in the proximal promoter (Cypess et al., 2011). In hematopoietic stem cells, p53 activates NDN transcription via a putative p53‐binding sequence at +43 in human NDN promoter (Liu, Elf, et al., 2009). Unliganded thyroid hormone receptors (apoTRs) activate Ndn transcription through a negative thyroid hormone response element (+88) downstream of the Ndn transcription start site, whereas the thyroid hormone‐liganded receptor represses Ndn transcription (Nygard et al., 2006). Some of these transcription factors are also necdin‐binding proteins as described later. Therefore, it is suggested that Ndn expression is controlled via positive and negative feedback loops.

### 2.5 Necdin as a transcription factor

The N‐terminal 100 aa domain of necdin is rich in proline and acidic aa. Protein regions rich in these aa are often seen in transactivation domains of transcription factors. In fact, this domain (aa 1–82) induces transactivation in the yeast GAL4 system (Taniura et al., 1998). Furthermore, necdin directly binds to specific DNA sequences rich in guanosine (G), termed GN boxes (Matsumoto et al., 2001) (Figure 4b). Necdin controls c‐Myc gene transcription by binding directly to a presumptive GN box motif in mouse c‐Myc P1 promoter. Necdin suppresses c‐Myc gene transcription in the presence of Sp1, a transcription factor that activates c‐Myc expression via the GN box, whereas necdin activates the c‐Myc expression in the absence of Sp1. Necdin‐mediated activation of c‐Myc expression via the P1 promoter GN box is antagonized by cystin, a cilia‐associated protein implicated in autosomal recessive polycystic kidney disease (Wu et al., 2013). Necdin also increases c‐Myc expression in mouse mammary tumor cell lines (Lee et al., 2015). In hepatic stellate cells implicated in the pathogenesis of liver fibrosis, necdin transactivates the Wnt10b gene by interacting with a putative GN box in its proximal promoter (Zhu et al., 2010).

## 3 GENOMIC IMPRINTING

### 3.1 Chromosomal localization

**FIGURE 5:**
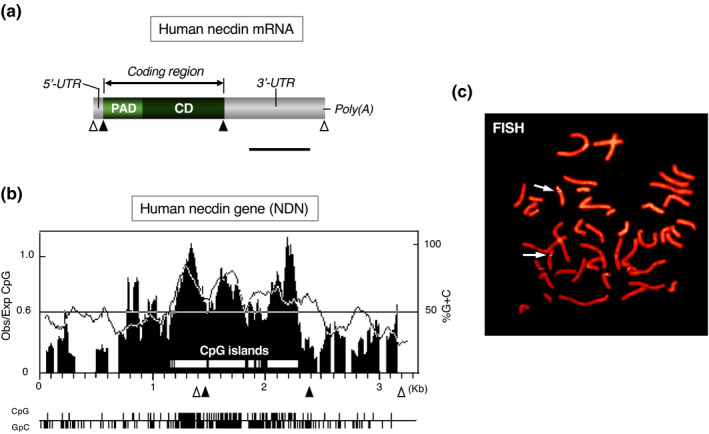
Human necdin gene NDN is a mono‐exonic gene containing CpG islands. (a) Structure of human necdin mRNA. Human necdin mRNA consists of the coding region (PAD, CD), 5'‐, 3'‐untranslated regions (UTR), and poly(A) tail. Open triangles, transcription start/stop sites and filled triangles, translation start/stop sites. Scale bar, 0.5 kb. (b) CpG islands in human NDN. Values for Obs/Exp CpG (vertical lines) and %G + C (dotted line) are plotted in the graph. The positions are numbered in kb. The regions of Obs/Exp CpG over 0.6 and %G + C over 50 are classified as CpG islands (open horizontal bars in the graph). Positions of CpG and GpC dinucleotide are below the graph. (c) Chromosomal localization of NDN. Human metaphase chromosomes were analyzed by fluorescence in situ hybridization (FISH) with human NDN probe. Chromosomes were stained with propidium iodide. The arrows point to twin‐spot signals on chromosome 15q11.2‐q12. Illustrations (a‐c) based on Nakada et al. (1998)

**FIGURE 6:**
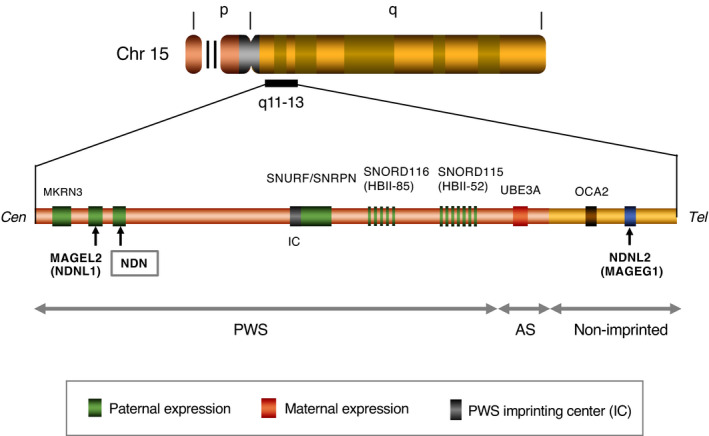
NDN and its homologous genes are located on human chromosome 15q11‐13. Human chromosome (Chr) 15q11–13 contains NDN and its homologous genes NDNL2 (MAGEG1) and MAGEL2 (NDNL1). In Chr 15q11–13, NDN and MAGEL2 are located in the imprinted region where imprinted genes are clustered, whereas NDNL2 is in the nonimprinted region. Representative imprinted genes are shown. Abbreviations: PWS, Prader–Willi syndrome and AS, Angelman syndrome. Note the locations of NDN and MAGEL2 in the PWS region. Not drawn to scale. Illustration based on Boccaccio et al. (1999), Lee et al. (2000) and Chibuk et al. (2001)

Human necdin mRNA comprises a coding region (963 nucleotides) and 3'‐, 5'‐untranslated regions (~940 nucleotides) (Nakada et al., 1998) (Figure 5a). Human NDN is a small intronless gene that contains CpG‐rich DNA regions known as CpG islands, where the frequency of the CpG sequence is higher than other regions (Figure 5b). CpG islands are located in the NDN region extending over the proximal 5' flanking sequence and the protein‐coding region. Expression of genes containing CpG islands is often regulated by DNA methylation. FISH analysis reveals that NDN is located on chromosome 15q11.2‐q12 (Figure 5c). This locus is included in the region responsible for PWS, a genomic imprinting‐associated neurodevelopmental disorder (Jay et al., 1997; MacDonald & Wevrick, 1997; Sutcliffe et al., 1997) (Figure 6). In addition to NDN, two NDN‐homologous genes MAGEL2 (NDNL1) and NDNL2 (MAGEG1, NSMCE3) are located on chromosome 15q12‐13 (Boccaccio et al., 1999; Chibuk et al., 2001; Lee et al., 2000). These three genes, designated here as NDN subfamily, are highly homologous to each other and presumedly emerged by gene duplication of an ancestral gene. On the other hand, mouse Ndn subfamily consisting of necdin (Ndn), Magel2 (Ndnl1) and Ndnl2 (Mageg1, Nsmce3) are located in the 7C region of mouse chromosome 7, a region of conserved synteny to human 15q11.2‐q12 (Boccaccio et al., 1999; Chibuk et al., 2001; Watrin et al., 1997). The genomic organization and the regulatory mechanism of imprinted gene expression are highly conserved in the syntenic regions, suggesting that these features have evolved in the lineage of placental mammals (Rapkins et al., 2006).

### 3.2 Paternal gene expression

In addition to NDN and MAGEL2, paternally expressed genes such as MKRN3, SNURF/SNRPN, SNORD116 and SNORD115 are located in the PWS region (Horsthemke & Wagstaff, 2008) (Figure 6). These genes share a common regulatory element known as the PWS imprinting center (PWS‐IC), which spans the major promoter and first exon 1 of SNURF/SNRPN. Maternal NDN allele undergoes hypermethylation of its promoter CpG islands, whereas the paternal NDN allele is located in a transcriptionally active domain embedded in the hyperacetylated histone‐rich chromatin (Lau et al., 2004). These features indicate that NDN is maternally imprinted and expressed only from the paternal allele through PWS‐IC‐dependent regulation. PWS‐IC is indispensable for the regulation of Ndn imprinting, but not for the spatio‐temporal regulation of Ndn expression in mice (Watrin et al., 2005), suggesting that PWS‐IC specifically controls the methylation status of NDN and other maternally imprinted genes. The mouse orthologs of paternally expressed PWS genes (Snrpn, Ipw, Ndn, Magel2, Mkrn3 and Snord116) are expressed in brain regions such as the hypothalamus, pituitary, forebrain and hindbrain during the embryonic period (Lee et al., 2003). Among these genes, Snrpn, Ipw and Ndn are expressed at relatively high levels throughout the mouse brain, whereas Magel2, Mkrn3 and Snord116 are preferentially expressed in specific brain regions. The fact that these imprinted genes are expressed predominantly in the embryonic brain suggests that these paternally expressed genes contribute to normal brain development in a parent‐of‐origin‐specific manner.

## 4 GENE FAMILY

### 4.1 MAGE family

**FIGURE 7:**
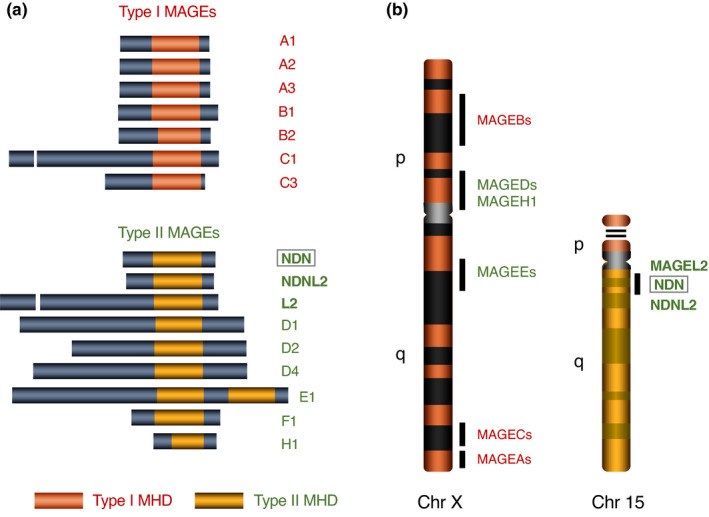
MAGE family proteins share conserved homology domains. (a) Human MAGE family proteins. MAGE family members containing conserved 160–170 aa domains (MAGE homology domain, MHD) are classified into two types based on aa similarities of MHDs: Type I MAGE family consists of the subfamilies MAGEA (MAGEAs), MAGEB (MAGEBs) and MAGEC (MAGECs); type II MAGE family of the subfamilies NDN (NDN, NDNL2, MAGEL2), MAGED subfamily (MAGEDs), MAGEE subfamily (MAGEEs), MAGEF1 and MAGEH1. Representative subfamily members are shown. Adapted from Barker and Salehi (2002). (b) MAGE family genes located on Chr X and Chr 15. Most MAGE family genes are located on Chr X, the NDN subfamily (NDN, NDNL2, MAGEL2) on Chr 15 and MAGEF1 on Chr 3 (not shown). Adapted from Chomez et al. (2001)

Necdin shares a homologous domain with MAGE family proteins (De Backer et al., 1995; De Plaen et al., 1994, 1999; Florke Gee et al., 2020). The MAGE genes have been originally identified as genes encoding precursors of tumor rejection antigens recognized by cytolytic T lymphocytes (van der Bruggen et al., 1991). These genes are expressed only in cancer cells and male germ cells (Chomez et al., 2001; Florke Gee et al., 2020; Lee & Potts, 2017; Weon & Potts, 2015), thus referred to as cancer–testis expression. The MAGE family comprises 36 members (humans) and 28 members (mouse), which share a large homology region (Chomez et al., 2001). The MAGE homology domain of necdin is included in the conserved domain. The MAGE family proteins are divided into two groups (type I and type II) based on sequence similarities of their homology domains (Barker & Salehi, 2002) (Figure 7a). Human type I MAGE genes including MAGEA, B and C subfamilies are expressed in transformed cells and germ cells but not in normal somatic cells. Mice lack a subfamily corresponding to human MAGEC subfamily, implying that this subfamily has emerged after divergence of primate and rodent lineages.

In contrast to type I MAGE members, type II MAGE members are expressed in differentiated cells including neurons and skeletal muscle (Barker & Salehi, 2002; Chomez et al., 2001; Kuwajima et al., 2004; Uetsuki et al., 1996). Relative mRNA expression levels in mouse embryonic brain and P19 EC cell‐derived neurons are as follows: necdin, Maged1 > Magee1, Mageh1 > Magel2, Ndnl2 > Magea, Mageb (undetectable) (Aizawa et al., 2011). Most MAGE genes are located on chromosome X, whereas NDN subfamily members (NDN, MAGEL2, NDNL2) are on chromosome 15 (Figure 7b). The MAGEF1 gene is located on chromosome 3 and expressed in the human brain (Stone et al., 2001). Noteworthily, human MAGE family genes located on chromosomes X and 15q11‐12 are monoallelically expressed, because random chromosome X inactivation and genomic imprinting, both of which are typical DNA methylation‐dependent inactivation processes, have co‐evolved in the lineage of placental mammals (Reik & Lewis, 2005). Accordingly, among human MAGE family genes only NDNL2 and MAGEF1 are presumedly expressed in a biallelic manner.

Many pseudogenes related to functional MAGE gene family are found in the human and mouse genomes. For example, 19 probable MAGE‐related pseudogenes are located on human chromosome X (Chomez et al., 2001). Moreover, a human necdin pseudogene is located on chromosome 12q14‐q21.1 (Nakada et al., 2000). In contrast to human MAGEF1, mouse Magef1 is presumptively an expressed pseudogene as judged from its cDNA sequence. Consequently, part of rapidly diversified MAGE genes may have lost their functions as pseudogenes during the evolution of placental mammals.

### 4.2 Evolution of MAGE family

Although mammalian MAGE gene family consists of many members, nonmammalian vertebrates such as fish (zebrafish, pufferfish) (Bischof et al., 2003) and chicken (Lopez‐Sanchez et al., 2007) possess single MAGE genes. In nonplacental mammals, the platypus (*Ornithorhynchus anatinus*), an extant species of monotremes, has only one MAGE gene, whereas marsupials such as the opossum (*Monodelphis domestica*) and Tasmanian devil (*Sarcophilus harrisii*) have two MAGE genes (De Donato et al., 2017; Katsura & Satta, 2011). The two opossum MAGE genes modoMAGEL1 and modoMAGEL2 are located on chromosomes X and 8, respectively. Noteworthily, modoMAGEL1 is coded by ~11 exons and modoMAGEL2 by single exon, suggesting that modoMAGEL2 is a processed gene derived from modoMAGEL1 (Katsura & Satta, 2011). This hints a similar process in placental MAGE gene evolution as described below. These nonplacental MAGE proteins display the highest homology to placental NDNL2 (NSMCE3) among mammalian MAGE members. As nonplacental vertebrates lack NDN orthologs, NDN has most likely emerged in placental mammals through MAGE gene diversification.

**FIGURE 8:**
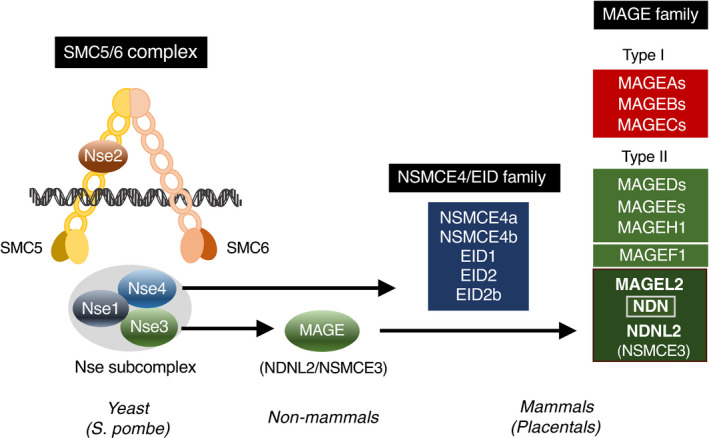
Mammalian MAGE family genes are homologous to the Nse3 component of the SMC5/6 complex. MAGE family members in mammals (Placentals) are homologous to Nse3, a non‐SMC element of the SMC5/6 complex. Animal species including invertebrates and nonmammalian vertebrates (nonmammals) possess only single MAGE (NDNL2/NSMCE3) genes. The fission yeast (*S. pombe*) SMC5/6 complex is composed of SMC5/6 and non‐SMC elements 1–4 (Nse1‐4). Note the NSMCE4/EID family diversified in placental mammals

In the fission yeast (*Schizosaccharomyces pombe*), Nse3 (non‐SMC element 3), a component of the subcomplex of SMC (structural maintenance of chromosomes) 5/6 complex, is homologous to mammalian MAGE family (Sergeant et al., 2005). This suggests that MAGE family genes including mammalian necdin have evolved from an Nse3‐like gene in an ancestor of placental mammals (Figure 8). Noteworthily, yeast Nse4, an Nse3‐binding partner, has been also diversified in mammals into five members of the NSE4/EID family (Nse4a, Nse4b, EID1, EID2, EID2b) (Guerineau et al., 2012). The fact that the SMC5/6 complex contributes to DNA repair and DNA damage response in yeasts (Lehmann, 2005) suggests that nonmammalian MAGE (NDNL2, NSMCE3) genes are also involved in DNA damage‐related processes as a component of the SMC5/6 complex.

Although nonplacental single‐type MAGE (NSMCE3) genes are multi‐exonic (9–11 exons in coding regions), NDNL2 (NSMCE3) genes in placental mammals are mono‐exonic (De Donato et al., 2017; Katsura & Satta, 2011; Lopez‐Sanchez et al., 2007). This suggests that placental mono‐exonic NDNL2 (NSMCE3) genes are processed genes derived from an ancestral multi‐exonic MAGE (NSMCE3) gene in a manner similar to the evolution of opossum MAGE genes as mentioned above. NDN and MAGEL2 in the imprinted gene‐clustered region may have arisen through duplication of NDNL2 (NSMCE3) located in the proximal region. In addition to the NDN subfamily genes, other type II MAGE genes such as MAGEF1, MAGEH1 and MAGEEs are also mono‐exonic (De Donato et al., 2017; Katsura & Satta, 2011), suggesting that these MAGEs have emerged in a manner similar to the NDN subfamily. Thus, emergence of the NDN subfamily may be a key event of the MAGE gene evolution.

### 4.3 Neural expression of nonmammalian MAGE

In the fruit fly (*Drosophila melanogaster*), MAGE is highly expressed in neural stem cells (neuroblasts), neuronal progenitor cells (ganglion mother cells) and postmitotic neurons at larval and pupal stages of postembryonic neurogenesis (Nishimura et al., 2007). In adult flies, MAGE is expressed in the brain (mushroom body, optic lobe), retina, flight muscle and ovaries. In the zebrafish (*Danio rerio*), MAGE is expressed in the nervous system including medial telencephalon, optic tectum, cerebellum and retina (Bischof et al., 2003). In the chicken (*Gallus gallus*), MAGE is highly expressed in the nervous system including the retina, spinal cord and dorsal root ganglia during development (Lopez‐Sanchez et al., 2007). The expression patterns of nonmammalian single‐type MAGEs suggest that nonmammalian MAGEs play certain roles in neuronal development. Functional and evolutionary implications of these nonmammalian MAGEs will be discussed later in this review.

## 5 GENE TARGETING

### 5.1 Paternal allele mutant mice

**FIGURE 9:**
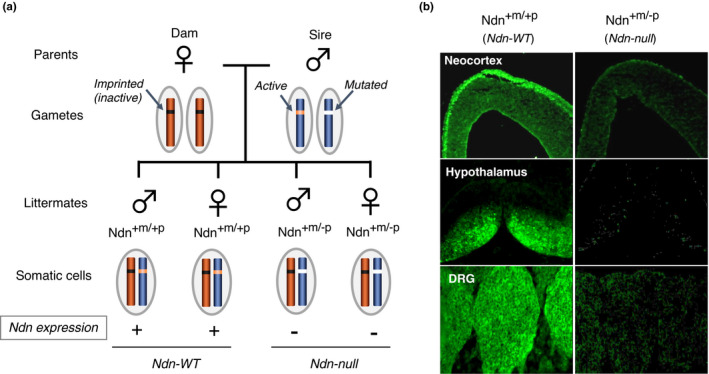
Generation of paternal Ndn‐mutant mice. (a) Generation of Ndn‐null mice. In gametes, maternal Ndn on Chr 7 is imprinted (inactive), whereas paternal Ndn is nonimprinted (active). Littermates carrying genotypes Ndn^+m/−p^ (paternal Ndn mutated) and Ndn^+m/+p^ (both wild‐type Ndn) are phenotypically null mutant (Ndn‐null) and wild type (Ndn‐WT), respectively. (b) Necdin immunohistochemistry. Paternal Ndn‐mutated mouse embryos (Ndn^+m/−p^, Ndn‐null) fail to express necdin in neural tissues where necdin is highly expressed in wild‐type mice (Ndn^+m/+p^, Ndn‐WT) as analyzed by immunohistochemistry. DRG, dorsal root ganglion. Adapted from Kuwako et al. (2005)

Ndn‐targeted mice are suitable experimental systems for analyzing normal functions of necdin. As mouse Ndn is maternally imprinted and expressed only from the paternal allele, littermates carrying the mutated Ndn allele inherited from their sire (Ndn^+m/−p^) are Ndn‐null irrespective of wild‐type allele inherited from their dam (Figure 9). When a heterozygous sire (Ndn^+m/−p^) is crossed with a wild‐type dam (Ndn^+m/+p^), half of the littermates are predictedly Ndn‐null (Ndn^+m/−p^), whereas the other half are wild type (Ndn^+m/+p^). This feature is advantageous to well‐controlled experiments with adequate numbers of Ndn‐null and wild‐type littermates.

Mice carrying the paternally inherited Ndn‐mutant allele (Ndn^tm1Stw^) exhibit early postnatal lethality due to respiratory insufficiency (Gerard et al., 1999). The rate of postnatal lethality, which is affected by genetic backgrounds, is the highest in the C57BL/6 inbred background. Mice surviving the critical neonatal period are indistinguishable from the wild‐type littermates in their postnatal development. The neonatal lethality of variable penetrance is also observed in another Ndn‐mutant mouse strain (Ndn^tm1Mus^) (Muscatelli et al., 2000). Intriguingly, the maternal Ndn allele is stochastically expressed at an extremely low level in the absence of paternal Ndn allele (Rieusset et al., 2013). This type of Ndn expression reduces birth lethality and severity of breathing deficiency. In contrast, there are other strains of paternal Ndn‐mutated mice that show no neonatal lethality (Kuwako et al., 2005; Tsai et al., 1999). For example, paternal Ndn‐mutated mice (Ndn^tm1Ky^) show no obvious phenotypes on the background of ICR (CD‐1), a highly prolific outbred strain (Kuwako et al., 2005). However, these mutant mice exhibit various types of abnormalities at cellular and molecular levels as described below.

### 5.2 Neuronal phenotypes

#### 5.2.1 Cerebrum

Morphological and biochemical abnormalities in various types of neurons are found throughout the nervous system of Ndn‐null mice (Table 1). In the neocortex of embryonic Ndn‐null mice (Ndn^tm1Ky^), the proliferative and apoptotic populations of NSPCs are significantly increased at the ventricular zone (Minamide et al., 2014). In the ganglionic eminence, a transitory forebrain structure at the embryonic stages, the populations of proliferating and apoptotic cells increase in Ndn‐null mice (Ndn^tm1Ky^) (Huang et al., 2013), whereas the population of GABAergic neurons decreases (Kuwajima et al., 2006). In the neocortex of Ndn‐null mice (Ndn^tm1Ky^) at the neonatal stage, the population of GABAergic interneurons decreases significantly (Kuwajima et al., 2010). These findings suggest that necdin promotes the differentiation and migration of GABAergic interneurons during forebrain development. Interestingly, neocortical levels of ATP and mitochondrial gene expression are markedly reduced in Ndn‐null mice (Ndn^tm1Ky^) from the embryonic to late adult stages (Hasegawa et al., 2016), implying that necdin promotes mitochondrial biogenesis in neocortical neurons throughout the lifetime.

**TABLE 1:** Neuronal phenotypes in vivo of Ndn‐null mice

Region	Ndn‐null phenotype (age)	Reference
Cerebrum		
Neocortex	Increased populations of proliferating and apoptotic NSPCs (E14.5)	Minamide et al. (2014)
	Decreased GABAergic neuron number (E17.5, P0)	Kuwajima et al. (2010)
	Reduced levels of ATP (E14.5, P0, P17M) and mitochondrial markers (P17W, P60W)	Hasegawa et al. (2016)
Ganglionic eminences	Increased populations of proliferating and apoptotic NSPCs (E14.5)	Huang et al. (2013)
	Decreased GABAergic neuron number (E14.5)	Kuwajima et al. (2006)
Hypothalamus		
Preoptic area	Decreased GnRH neuron number (adult)	Muscatelli et al. (2000)
	Decreased GnRH neuron number (E13.5), defective axon projection (E17.5)	Miller et al. (2009)
	Increased GABAergic neuron number (E17.5, P0)	Kuwajima et al. (2010)
Arcuate nucleus	Increased FoxO1 acetylation (P28)	Hasegawa et al. (2012)
Paraventricular nucleus	Decreased oxytocin neuron number (adult)	Muscatelli et al. (2000)
	Reduced TRH mRNA expression (P30)	Hasegawa et al. (2012)
Suprachiasmatic nucleus	Reduced expression of clock genes (P4M‐P6M)	Lu et al. (2020)
Midbrain		
Substantia nigra	Disorganized nigro‐striatal pathway (E15.5)	Lee et al. (2005)
	Decreased dopaminergic neuron number, reduced mitochondrial levels (P60W)	Hasegawa et al. (2016)
Locus coeruleus	Decreased electrophysiological activity of noradrenergic neurons (organotypic slice culture)	Wu et al. (2020)
Hindbrain		
Cerebellum	Increased granule neuron apoptosis in cerebellar cortex (P6)	Kurita et al. (2006)
Medulla/Pons	Abnormal neuronal activity in respiratory rhythm‐generating center (E18.5)	Ren et al. (2003)
	Cytoarchitectural abnormalities in respiratory rhythm‐generating center (E18)	Pagliardini et al. (2005)
	Abnormal projection of serotonergic neurons (E12.5, E15.5)	Lee et al. (2005)
	Morphological and functional abnormalities of serotonergic neurons (E16.5, P2, P30)	Zanella et al. (2008)
	Functional and biochemical abnormalities of serotonergic neurons (E11.5‐P60)	Matarazzo et al. (2017)
Other regions		
Spinal cord	Cytoarchitectural abnormalities (E10‐E18)	Pagliardini et al. (2005)
	Increased apoptosis (E13.5), decreased motor neuron number (E17.5)	Aebischer et al. (2011)
Dorsal root ganglia	Increased apoptosis (E12.5), decreased sensory neuron number (P0)	Kuwako et al, (2005)
	Increased apoptosis (E11.5, E12.5), decreased sensory neuron number (E13.5)	Andrieu et al. (2006)
Sympathetic ganglion	Abnormal neuronal migration, impaired axonal outgrowth, increased apoptosis (E18.5)	Tennese et al. (2008)
Retina	Defective axonal fasciculation of retinal ganglion cells (E18.5)	Lee et al. (2005)

Abbreviations: E#, embryonic day #; NSPCs, neural stem/progenitor cells; P#, postnatal day #; P#M, postnatal month #; P#W, postnatal week #.

#### 5.2.2 Hypothalamus

In the hypothalamus of Ndn‐null mice (Ndn^tm1Mus^) that survive the neonatal critical period, the populations of neurons producing oxytocin and gonadotropin‐releasing hormone (GnRH) are significantly reduced at the adult stage (Muscatelli et al., 2000). In Ndn‐null mice (Ndn^tm2Stw^), the number of hypothalamic GnRH neurons is reduced, and extension of GnRH‐immunopositive axons to the median eminence is impaired (Miller et al., 2009). In the arcuate nucleus, acetylated FoxO1 levels are increased in Ndn‐null mice (Ndn^tm1Ky^), whereas TRH (thyroid hormone‐releasing hormone) mRNA levels in the paraventricular nucleus are reduced (Hasegawa et al., 2012). Ndn‐null mice (CRISPR‐Cas9 system) display abnormal expression patterns of clock genes in the suprachiasmatic nucleus known as a hypothalamic circadian rhythm‐generating center (Lu et al., 2020).

#### 5.2.3 Midbrain and hindbrain

In the midbrain of Ndn‐null mice (Ndn^tm1Ky^), the number of dopaminergic neurons is reduced at the substantia nigra in late adulthood (Hasegawa et al., 2016). In the locus coeruleus, spontaneous electrophysiological activity of noradrenergic neurons is significantly reduced in Ndn‐null mice (Ndn^tm1Ky^) (Wu et al., 2020). In the cerebellum of Ndn‐null mice (Ndn^tm1Ky^), apoptotic cell populations in the external and internal granule layers are significantly increased (Kurita et al., 2006). Ndn‐null mice (Ndn^tm2Stw^) suffering from respiratory insufficiency exhibit abnormal neuronal activities in the pre‐Botzinger complex, a putative respiratory rhythm‐generating center (Ren et al., 2003), where severe morphological abnormalities are found (Pagliardini et al., 2005). Moreover, the serotonergic system involved in respiratory control is impaired in Ndn‐null mice (Ndn^tm1Mus^) (Matarazzo et al., 2017; Zanella et al., 2008).

#### 5.2.4 Other regions

In the spinal cord, a significant loss of motoneurons is observed in Ndn‐null mice (Ndn^tm1Mus^) during the period of programmed cell death (Aebischer et al., 2011). In the dorsal root ganglia (DRG) of Ndn‐null mice (Ndn^tm1Ky^, Ndn^tm1Mus^), the population of sensory neurons is significantly reduced, whereas the apoptotic cell population is increased (Andrieu et al., 2006; Kuwako et al., 2005), indicating that necdin promotes survival of neurotrophin‐dependent sensory neurons. In the sympathetic ganglia, Ndn‐null mice (Ndn^tm2Stw^) show abnormal migration of sympathetic neurons accompanied by impaired axonal outgrowth (Tennese et al., 2008). Axonal abnormalities are also observed in retinal ganglion cells, serotonergic and catecholaminergic neurons in Ndn‐null mice (Ndn^tm2Stw^) (Lee et al., 2005). These findings suggest that necdin is indispensable for axonal development.

### 5.3 Physical and behavioral phenotypes

The neonatal respiratory distress mentioned above is the most striking phenotype of Ndn‐null mice. Additionally, Ndn‐null mice display a variety of physical and behavioral abnormalities. Ndn‐null mice show skin picking and improved performance in the Morris water maze test, indicating increased spatial learning and memory (Muscatelli et al., 2000). Furthermore, Ndn‐null mice exhibit phenotypes that are largely explained by neuronal abnormalities: increased pentylenetetrazole (PTZ)‐induced seizure susceptibilities due to reduced populations of cortical GABAergic inhibitory neurons (Kuwajima et al., 2010), low body temperatures due to central hypothyroidism caused by functional abnormalities of the hypothalamic neurons (Hasegawa et al., 2012), increased pain thresholds due to reduced sensory neuron populations in the DRG (Kuwako et al., 2005), abnormal circadian rhythm‐related behaviors caused by abnormal clock gene expression in neurons at the hypothalamic suprachiasmatic nucleus (Lu et al., 2020), transient hypotonia (Wu et al., 2020) and blunt respiratory responses to hypercapnia due to abnormal activities of locus coeruleus neurons (Wu et al., 2020). Although mutant mice (Ndn^tm1Ky^) exhibit no apparent abnormalities in food‐related behaviors such as hyperphagia, adipose tissues in Ndn‐null mice are markedly enlarged owing to preadipocyte hyperproliferation when fed with high calorie diets during juvenile and adult periods (Fujiwara et al., 2012).

## 6 CELL FUNCTION I: SUPPRESSION OF MITOSIS

### 6.1 Transfected cells

**FIGURE 10:**
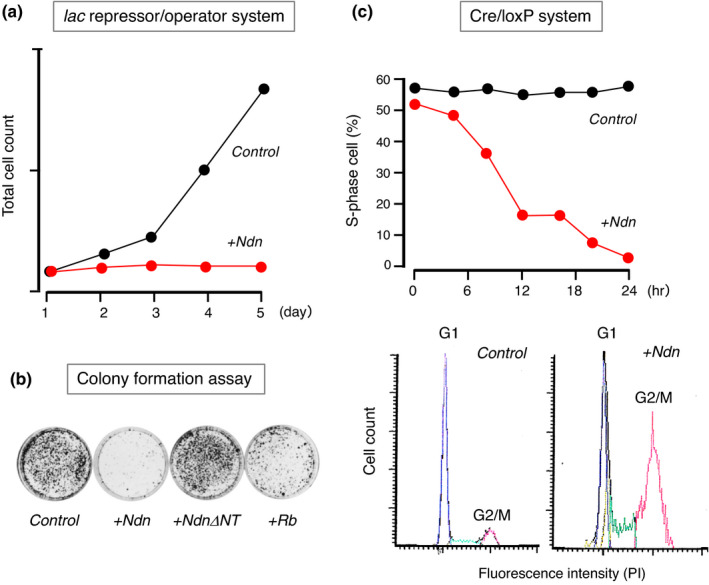
Ectopic expression of necdin strongly suppresses cell proliferation. (a) Conditional expression in *lac* repressor–operator system. NIH3T3 cells were stably transfected with necdin cDNA in the lac repressor–operator vector and cultured in the absence (Control) or presence (Ndn+) of the expression inducer IPTG. The number of cells at each time point was determined by colorimetry. For experimental details, see Hayashi et al. (1995). (b) Transfected SAOS2 cells. SAOS‐2 cells were transfected with empty vector (Control) and cDNA for necdin (+Ndn), necdin N‐terminal 109 aa deletion mutant (+NdnΔNT) or retinoblastoma protein (+Rb). Transfectants were grown for 14 days for selecting stable transfectants, fixed and visualized by crystal violet staining. For experimental details, see Taniura et al. (1998). (c) Conditional expression in Cre/loxP system. NIH3T3 cells were stably transfected with necdin expression vector carrying loxP sites. Transfectants were infected with Cre recombinase‐expressing adenovirus, incubated with bromodeoxyuridine (BrdU) for 60 min and fixed at each time point for BrdU‐immunopositive cell count (S‐phase cells) (C, upper panel). For cell cycle analysis, transfectants were stained with propidium iodide (PI) 24 hr after adenovirus infection and analyzed by laser‐scanning cytometry (c, lower panels). Control, β‐galactosidase cDNA transfectant and +Ndn, necdin cDNA transfectant (Kawahara, K. & Yoshikawa, K., unpublished data)

Necdin is strongly expressed in neurons and skeletal muscle cells, both of which are typical postmitotic cells (Uetsuki et al., 1996), whereas most of transformed cell lines lack necdin expression (Aizawa et al., 1992). This leads to the experiments examining whether ectopic Ndn expression suppresses the proliferation of Ndn‐deficient cells. Conditional necdin expression in NIH3T3 cells using a eukaryotic lac repressor–operator expression system induces mitotic arrest without appreciable reduction in cell viability (Hayashi et al., 1995) (Figure 10a). Necdin strongly suppresses the colony formation of SAOS‐2 osteosarcoma cells, a cell line lacking retinoblastoma protein (Rb) (Taniura et al., 1998) (Figure 10b), indicating that necdin, unlike cyclin‐dependent kinase inhibitors such as p16 and p21, induces mitotic suppression in the absence of Rb. Necdin‐expressing SAOS‐2 cells show endoreduplication, a characteristic of G2/M‐arrested cells that fail to undergo cytokinesis (Taniura et al., 2005). Cre‐LoxP system‐mediated expression of necdin in NIH‐3T3 cells rapidly reduces the population of S‐phase cells (Figure 10c, upper panel). Ndn‐expressing cells exhibit a pattern characteristic of G2/M cell cycle arrest (Figure 10c, lower panel). These findings indicate that necdin exerts a potent antimitotic effect on proliferative cells.

### 6.2 Similarity to retinoblastoma protein

**FIGURE 11:**
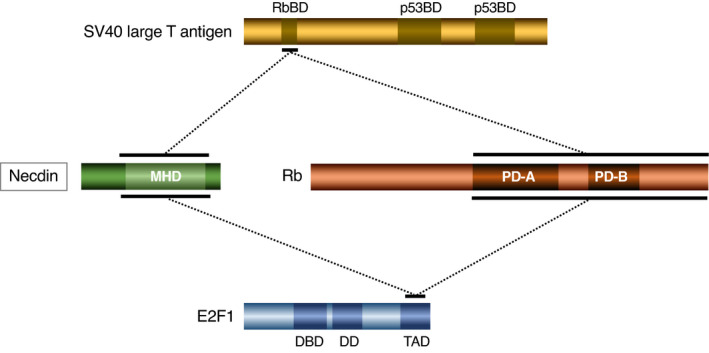
Necdin interacts with SV40 large T antigen and cellular transcription factor E2F1. The binding domains of simian virus 40 (SV40) large T antigen and E2F1 were determined by yeast two‐hybrid and in vitro binding assays using deletion mutants of individual proteins. Horizontal bar, binding domain and dotted line, interaction. Abbreviations: Rb, retinoblastoma protein; RbBD, Rb‐binding domain; p53BD, p53‐binding domain; MHD, MAGE homology domain; PD‐A, pocket domain‐A; PD‐B, pocket domain‐B; DBD, DNA‐binding domain; DD, dimerization domain; and TAD, transactivation domain. For experimental details, see Taniura et al. (1998)

DNA tumor virus oncoproteins such as SV40 (simian virus 40) large T antigen and adenovirus E1A bind to cellular tumor suppressor proteins such as Rb and p53. Necdin, like Rb, interacts with these viral oncoproteins (Taniura et al., 1998). Necdin binds to the N‐terminal Rb‐binding region of SV40 large T antigen (Figure 11). The necdin‐binding domains of SV40 large T antigen and adenovirus E1A contain the LxCxE motif. Interestingly, necdin interacts with the cellular LxCxE motif‐containing protein EID‐1 (EP300‐interacting inhibitor of differentiation 1) (Bush & Wevrick, 2008), which also interacts with Rb. These findings indicate that the target protein selectivity of necdin resembles that of Rb. The fact that necdin is a target of virus‐derived oncoproteins suggests that necdin, like the tumor suppressor proteins Rb and p53, suppresses cell division under physiological conditions.

### 6.3 Interaction with E2F1

E2F family members play key roles in the regulation of proliferation and differentiation in neuronal differentiation (Yoshikawa, 2000). Rb regulates the cell cycle progression by interacting with E2F: Unphosphorylated Rb binds E2F to suppress E2F1‐dependent cell cycle progression, whereas phosphorylation of Rb by cyclin‐dependent kinases releases the suppression. Necdin binds to the C‐terminal transactivation domain of E2F1 and represses E2F‐dependent transactivation (Taniura et al., 1998) (Figure 11). Like Rb, necdin interacts with E2F1 and E2F4, but only E2F1 counteracts necdin‐induced mitotic arrest (Kobayashi et al., 2002). These properties of necdin resemble those of Rb. Noteworthily, the necdin‐homologous protein NDNL2 (MAGEG1, NSMCE3) interacts with E2F1, represses E2F1‐dependent transcription and suppresses E2F1‐induced apoptosis (Kuwako et al., 2004). Interestingly, chicken MAGE (NSMCE3) also suppresses E2F1‐induced apoptosis (Lopez‐Sanchez et al., 2007). These findings suggest that the E2F1‐interacting property is evolutionarily conserved among MAGE gene family.

### 6.4 Neural stem/progenitor cells

**FIGURE 12:**
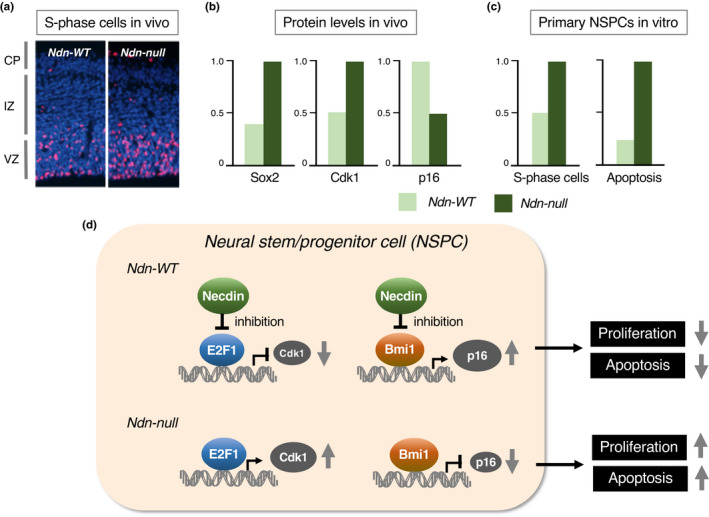
Necdin suppresses both proliferation and apoptosis of neural stem/progenitor cells. (a) S‐phase cells in the neocortex in vivo. Forebrain sections of E14.5 mouse embryos were prepared 4 hr after BrdU injection into pregnant mice. BrdU‐labeled cells (S‐phase cells) in the neocortex were detected by fluorescence immunohistochemistry for BrdU. Chromosomal DNA was stained with Hoechst 33342. See Figure 2 for abbreviations. (b) Neocortical levels of Sox2, Cdk1 and p16 in vivo. Neocortical protein levels at E14.5 were analyzed by immunoblotting for individual proteins and quantified by densitometry. (c) Proliferation and apoptosis of primary neural stem/progenitor cells (NSPCs). Primary NSPCs prepared from E14.5 mice were cultured in the presence of growth factors, incubated with BrdU for 4 hr, immunostained for BrdU and analyzed for BrdU‐immunopositive cell count (S‐phase cells). Apoptotic cells (Apoptosis) were analyzed by TUNEL (terminal deoxynucleotidyl transferase dUTP nick end labeling). For experimental details (a‐c), see Minamide et al. (2014). (d) Diagram of interactions of necdin with E2F1 and Bmi1 in NSPCs. Necdin suppresses both E2F1‐dependent Cdk1 transactivation and Bmi1‐dependent p16 repression. Ndn‐null NSPCs show increased rates of proliferation and apoptosis

Excitatory neurons in the neocortex differentiate from NSPCs located at the ventricular zone and migrate radially into the marginal zone where they form the cortical plate (Paridaen & Huttner, 2014). Proliferative cell populations at the ventricular zone are significantly increased in Ndn‐null mice (Minamide et al., 2014) (Figure 12a). In the neocortex in vivo of Ndn‐null embryos, expression levels of the stem cell marker Sox2 and the cell cycle‐promoting cyclin‐dependent kinase Cdk1 (Cdc2) are increased, whereas expression of p16 (p16Ink4a), a cyclin‐dependent kinase inhibitor, is diminished (Figure 12b). These findings suggest that necdin suppresses the proliferation and apoptosis of neural stem/progenitor cells by both suppressing Cdk1 expression and promoting p16 expression. Neocortical NSPCs prepared from Ndn‐null embryos also show increased rates of proliferation and apoptosis in vitro (Figure 12c). Necdin interacts with E2F1 and suppresses E2F1‐dependent transactivation of Cdk1 (Kurita et al., 2006), whereas Bmi1, a neural stem cell marker, suppresses p16 expression to promote cell proliferation (Park et al., 2004). Necdin interacts directly with Bmi1 and increases p16 expression (Minamide et al., 2014). Collectively, necdin suppresses the proliferation and apoptosis of neocortical NSPCs by interacting with E2F1 to suppress Cdk1 expression and with Bmi1 to increase p16 expression (Figure 12d).

**FIGURE 13:**
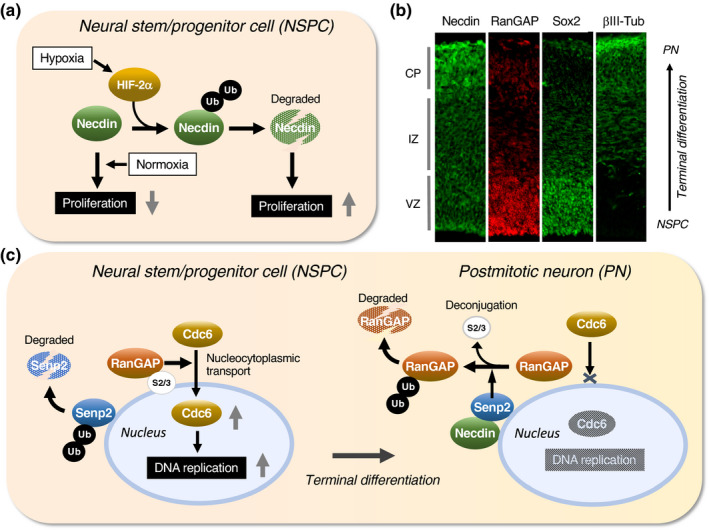
Necdin controls proliferation of neural stem/progenitor cells through protein modifications. (a) Diagram of ubiquitin‐dependent necdin degradation. In hypoxic conditions, stabilized HIF‐2α induces ubiquitination and subsequent proteasomal degradation of necdin. In normoxic conditions, necdin is stabilized and exerts its antimitotic effect. Illustration based on Huang et al. (2013). (b) Neocortical expression of cell marker proteins in vivo. Forebrain sections of mouse embryos at E14.5 were immunostained for necdin, RanGAP, Sox2 and βIII tubulin (βIII‐Tub), a postmitotic neuron marker (PN). See Figure 2 for abbreviations. (c) Diagram of nuclear transport system remodeling during terminal differentiation. RanGAP promotes nucleocytoplasmic transport of the DNA replication licensing factor Cdc6. During terminal differentiation, SUMO‐conjugated RanGAP undergoes Senp2‐mediated SUMO deconjugation followed by ubiquitin‐dependent degradation. This process blocks Cdc6 nuclear import and terminates DNA replication. Necdin promotes Senp2‐mediated RanGAP desumoylation. Ub, ubiquitin and S2/3, SUMO‐2/3. Illustrations (b, c) based on Fujiwara et al. (2016)

Primary NSPCs prepared from the ganglionic eminences of Ndn‐null mice show higher rates of proliferation and apoptosis than those from wild‐type mice (Huang et al., 2013). Hypoxia enhances proliferation of NSPCs and significantly reduces necdin levels. In hypoxic NSPCs, stabilized HIF2α (hypoxia‐inducible factor 2 α) interacts with necdin and promotes ubiquitin‐dependent degradation of necdin (Figure 13a), suggesting that necdin protein levels in NSPCs are regulated in an oxygen tension‐dependent manner to modulate the proliferation rate of NSPCs. RanGAP‐mediated nuclear transport system mediates cell cycle exit for terminal differentiation of neocortical NSPCs (Fujiwara et al., 2016). NSPCs located in the ventricular zone express high levels of Sox2 and RanGAP (Ran GTPase‐activating protein 1), a key regulator of the Ran GTP‐GDP cycle for nuclear transport (Figure 13b). The RanGAP levels are drastically reduced during neuronal terminal differentiation. In this process, SUMO (small ubiquitin‐like modifier)‐2/3‐conjugated RanGAP undergoes Senp2‐mediated desumoylation and subsequent ubiquitin‐dependent degradation. This process impedes the nuclear import of the DNA replication initiation factor Cdc6 and leads to mitotic termination (Figure 13c). Necdin interacts with both Senp2 and RanBP2, a nuclear pore component carrying E3 SUMO ligase activity, and promotes Senp2‐mediated desumoylation of RanGAP (Fujiwara et al., 2016). Interestingly, Senp2 also catalyzes CREB desumoylation and suppresses Ndn expression (Liang et al., 2019), suggesting that Ndn expression is controlled via a Senp2‐mediated negative feedback loop.

## 7 CELL FUNCTION II: SUPPRESSION OF APOPTOSIS

### 7.1 DNA damage response

**FIGURE 14:**
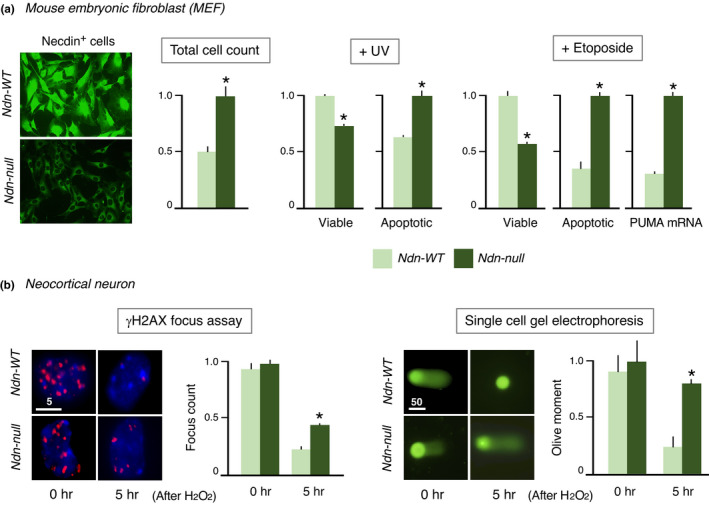
Necdin‐deficient cells are highly susceptible to DNA damage. (a) DNA damage‐induced apoptosis of mouse embryonic fibroblast (MEF). MEFs prepared from embryonal skins of Ndn‐WT and Ndn‐null mice at E13.5 were treated with ultraviolet light (UV, 100 J/m^2^, 15 min) or etoposide (50 μM, 24 hr) and analyzed 24 hr later by nuclear DNA staining for apoptotic cells or by trypan blue exclusion for viable cells. PUMA mRNA is quantified by quantitative reverse transcription PCR (qRT‐PCR) (Ibuki, M. & Yoshikawa, K., unpublished data). (b) DNA repair response in primary neurons. Primary neocortical neurons prepared from Ndn‐WT and Ndn‐null mice at E14.5 were treated with H_2_O_2_ (200 μM, 30 min) and analyzed 5 hr later for DNA repair activities by γH2AX‐immunopositive focus count (γH2AX focus assay) or by single cell gel electrophoresis (Olive moment). Each value represents mean ± *SEM* (*n* = 4). *p < .05. Numbers above scale bars in μm (Misawa, A. & Yoshikawa, K., unpublished data)

The DNA damage response is a key cellular mechanism that maintains the genome stability against DNA‐damaging insults (Giglia‐Mari et al., 2011). Various stimuli such as UV (ultraviolet) irradiation, ionizing radiation, genotoxic chemicals and reactive oxygen species induce double‐strand breaks of chromosomal DNA. These DNA damages are repaired by homologous recombination in proliferative cells and by nonhomologous end joining in postmitotic cells. Mouse embryonal fibroblasts (MEFs) express necdin at relatively high levels. Ndn‐null MEFs show higher proliferation rate than wild‐type MEFs (Figure 14a). In Ndn‐null MEFs treated with UV irradiation or the genotoxic topoisomerase II inhibitor etoposide, the viable cell population reduces, whereas the apoptotic cell population elevates. Expression of PUMA (p53 up‐regulated modulator of apoptosis) is markedly elevated in Ndn‐null MEFs, suggesting that endogenous necdin suppresses p53‐dependent PUMA expression. Furthermore, primary cortical neurons prepared from Ndn‐null mice show a significant delay in DNA repair after H_2_O_2_ treatment (Figure 14b). These data indicate that necdin promotes DNA repair and prevents DNA damage‐induced apoptosis.

### 7.2 Interaction with p53

**FIGURE 15:**
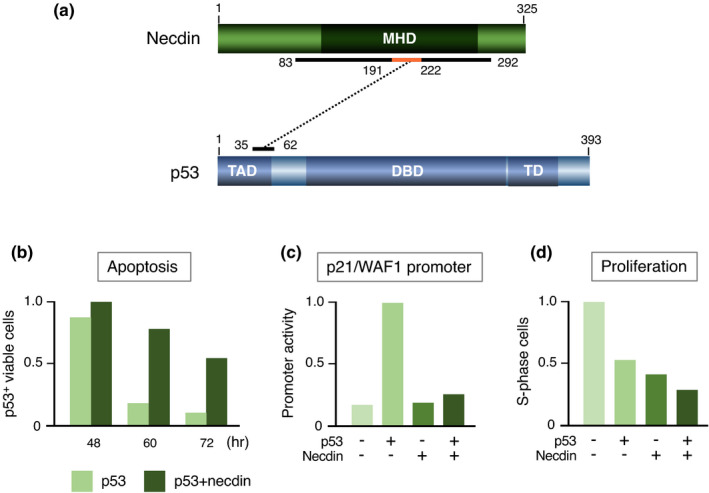
Necdin interacts directly with p53 to suppress p53‐mediated apoptosis. (a) Diagram of interaction between necdin and p53. The binding domains were determined by yeast two‐hybrid and in vitro binding assays using deletion mutants. Numbers, aa positions; p53‐interacting core domain in orange. Abbreviations: TAD, transactivation domain; DBD, DNA‐binding domain; and TD, tetramerization domain. For experimental details, see Taniura et al. (1999), Taniura et al. (2005). (b) p53‐mediated apoptosis assay. U2OS‐2 cells transfected with cDNAs for p53 and necdin were immunostained for p53, and p53‐immunopositive viable cells were counted at each time point by fluorescence microscopy. (c) p53‐dependent p21/WAF1 promoter assay. SAOS‐2 cells were transfected with a luciferase reporter vector carrying the p21/WAF1 promoter and combinations of cDNAs for necdin and p53. The luciferase activities were assayed by chemiluminometry. (d) Cell proliferation assay. HEK293 cells were transfected with combinations of cDNAs for necdin and p53, treated 36 hr later with BrdU for 2 hr and immunostained for BrdU‐immunopositive cell count (S‐phase cells). For experimental details (b‐d), see Taniura et al. (1999)

p53 plays a fundamental role in DNA repair to maintain genome stability by orchestrating a variety of DNA damage response processes (Hafner et al., 2019). Additionally, p53 induces apoptosis of damaged cells that fail to repair DNA. Necdin interacts with the N‐terminal transactivation domain of p53 (Taniura et al., 1999) (Figure 15a). p53 binds to the conserved domain of necdin but fails to interact with the aa 191–222 deletion mutant, indicating that this 32 aa domain is responsible for the interaction with p53 (Taniura et al., 2005). Functional analyses reveal that necdin inhibits p53‐dependent apoptosis (Figure 15b) and represses p53‐mediated transactivation (Figure 15c). In contrast, necdin fails to counteract p53‐induced mitotic suppression (Figure 15d), indicating that necdin and p53 suppress mitosis via distinct pathways.

### 7.3 p53‐mediated neuronal apoptosis

**FIGURE 16:**
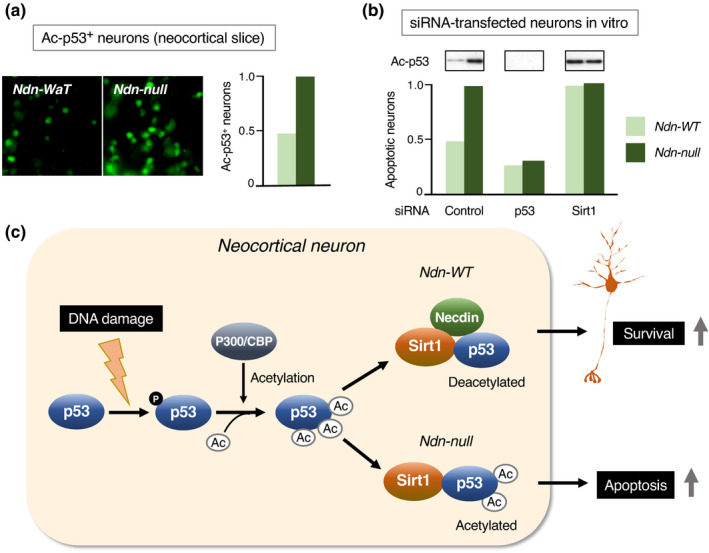
Necdin suppresses p53‐mediated apoptosis of neocortical neurons. (a) Acetylated p53 expression in neocortical neurons. Forebrain slices prepared from E14.5 mice were treated with H_2_O_2_ for 6 hr and immunostained for acetyl‐K373 p53 (Ac‐p53) (Ac‐p53^+^ neurons). (b) Effects of p53 and Sirt1 down‐regulation. Primary cortical neurons were prepared from E14.5 mice, transfected with small interfering RNA (siRNA) for control, p53 or Sirt1, and analyzed 24 hr later. For p53 acetylation, cell lysates were immunoblotted for Ac‐p53 (insets). For apoptosis, siRNA‐transfected neurons carrying apoptotic nuclei were counted. For experimental details (a, b), see Hasegawa and Yoshikawa (2008). (c) Diagram of DNA damage‐induced p53 acetylation regulated by necdin and Sirt1. DNA damage induces p53 phosphorylation (p) and p300/CBP‐mediated acetylation (Ac). Necdin promotes Sirt1‐mediated p53 deacetylation leading to neuronal survival

p53 plays a fundamental role in DNA damage‐induced apoptosis of postmitotic neurons (Miller et al., 2000). In response to DNA damage, p53 undergoes post‐translational modifications such as phosphorylation and acetylation (Hafner et al., 2019). Acetylated p53, which strongly promotes apoptosis of postmitotic neurons, is deacetylated by Sirt1 (sirtuin1), a mammalian ortholog of yeast Sir2 (silence information regulator 2) (Haigis & Sinclair, 2010). Necdin interacts with both p53 and Sirt1 to form a stable ternary complex in postmitotic neurons (Hasegawa & Yoshikawa, 2008). Acetylation of p53 is increased in Ndn‐null neocortical neurons under oxidative stress conditions (Figure 16a). Both p53 acetylation and p53‐induced apoptosis are enhanced in primary neocortical neurons prepared from Ndn‐null mice. The apoptotic cell population decreases in Ndn‐null neurons treated with p53 siRNA and increases in wild‐type neurons treated with Sirt1 siRNA (Figure 16b). The apoptotic rates are consistent with the acetylated p53 levels. These data suggest that necdin suppresses DNA damage‐induced neuronal apoptosis by promoting Sirt1‐mediated p53 deacetylation (Figure 16c).

### 7.4 E2F1‐mediated neuronal apoptosis

**FIGURE 17:**
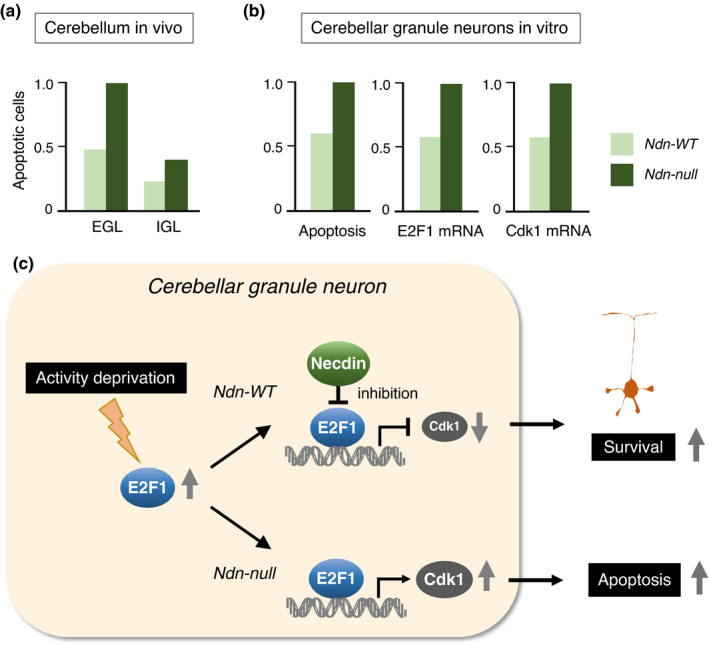
Necdin suppresses E2F1‐mediated apoptosis of cerebellar granule neurons. (a) Increased apoptosis in Ndn‐null cerebellum in vivo. Apoptotic cells in the cerebellum prepared from P6 mice were detected by TUNEL. EGL, external granule layer and IGL, internal granule layer. (b) E2F1 and Cdk1 mRNA levels in primary cerebellar granule neurons. Primary neurons were prepared from P6 mice, treated with low K^+^ medium for activity deprivation and analyzed for apoptotic cells by TUNEL (Apoptosis) and for E2F1 and Cdk1 mRNA levels by qRT‐PCR. For experimental details (a, b), see Kurita et al. (2006). (c) Diagram of necdin‐mediated inhibition of E2F1‐dependent neuronal apoptosis. Necdin suppresses E2F1‐dependent apoptosis of cerebellar granule neurons by inhibiting E2F1‐mediated Cdk1transactivation

E2F1 is a potent inducer of neuronal apoptosis (Yoshikawa, 2000). E2F1 increases Cdk1 (cyclin‐dependent kinase 1) expression and induces cell death by triggering premature entry into mitosis and consequent mitotic catastrophe (Castedo et al., 2002). Cerebellar granule neurons (CGNs) differentiate in vivo during the neonatal period of mouse development. In the cerebellum, necdin is strongly expressed in the internal granule layer where differentiated CGNs are enriched (Kurita et al., 2006). In the external granule layer where CGN precursors accumulate, the apoptotic cell population increases markedly in Ndn‐null mice (Figure 17a). Primary Ndn‐null CGNs show higher apoptotic rates than wild‐type CGNs when treated with low K^+^ concentration that induces activity deprivation. The mRNA levels of E2F1 and Cdk1 are also increased in Ndn‐null CGNs (Figure 17b). These data suggest that necdin suppresses transcriptional activity of E2F1, reduces E2F1‐mediated Cdk1 expression and attenuates E2F1‐induced apoptosis of CGNs under activity‐deprived conditions (Figure 17c).

## 8 CELL FUNCTION III: PROMOTION OF NEURONAL DIFFERENTIATION

### 8.1 Transfected cells

Since necdin expression is undetectable in transformed cell lines of neuroectodermal origin (Aizawa et al., 1992), these cells provide useful tools for analyzing the effects of necdin on neuronal differentiation. N1E‐115 neuroblastoma cells transfected with Ndn cDNA show differentiated phenotypes such as neurite outgrowth and synaptic marker protein expression (Kobayashi et al., 2002). PC12 pheochromocytoma cells transfected with Ndn cDNA exhibit accelerated neuronal differentiation in response to NGF (nerve growth factor) (Tcherpakov et al., 2002). Furthermore, ectopic expression of necdin in PC12 cells induces sustained phosphorylation of NGF‐stimulated TrkA (tropomyosin‐related kinase A) and MAPK (mitogen‐activated protein kinase) (Kuwako et al., 2005). Nogo‐A, a differentiation suppressing membrane protein, suppresses necdin‐induced neurite extension in PC12 cells by retaining necdin in the cytoplasm (Liu, Wang, et al., 2009). These findings indicate that ectopic necdin promotes neuronal differentiation of transformed cell lines.

### 8.2 Sensory neurons

Sensory neurons are afferent cells that transmit electrical signals of physical stimuli sensed by peripheral sensory receptors to the central nervous system. DRG (dorsal root ganglion) is a cluster of sensory neurons that require neurotrophins (neurotrophic factors) such as NGF for their differentiation and survival. NGF strongly increases necdin expression in sensory neurons prepared from mouse embryonic DRGs (Takazaki et al., 2002). Down‐regulation of necdin expression in DRG cultures leads to a marked reduction in the population of sensory neurons, suggesting that necdin promotes the differentiation and survival of NGF‐dependent DRG neurons.

**FIGURE 18:**
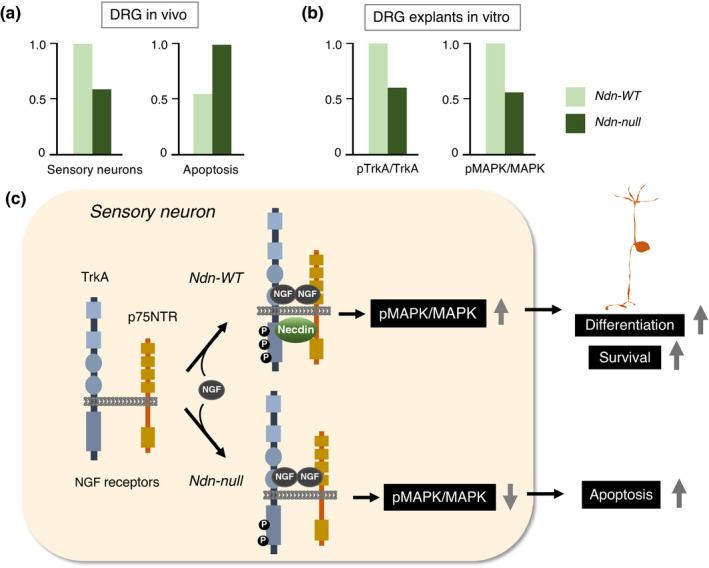
Necdin promotes differentiation of sensory neurons. (a) Viable and apoptotic neuron populations in DRG in vivo. Sensory neurons in mouse DRG at P0 were quantified by immunohistochemistry for the sensory neuron marker Substance P. Apoptotic cells in DRGs at E12.5 were detected by TUNEL. (b) Activation of TrkA and MAPK in DRG explants. DRG explants were prepared from E13.5 mice and treated with NGF for 15 min. Phosphorylated proteins (pTrkA, pMAPK) and total proteins (TrkA, MAPK) were analyzed by immunoblotting and quantified by densitometry for relative values (pTrkA/TrkA, pMAPK/MAPK). For experimental details (a, b), see Kuwako et al. (2005). (c) Diagram of necdin‐enhanced NGF‐receptor signaling in sensory neurons. Necdin interacts with both TrkA and p75, enhances NGF/TrkA/MAPK signal transduction and promotes the differentiation and survival of sensory neurons

Necdin binds to two classes of NGF transmembrane receptors p75NTR (Tcherpakov et al., 2002) and the receptor tyrosine kinase TrkA (Kuwako et al., 2005). In Ndn‐null DRG in vivo, the number of DRG sensory neurons decreases owing to enhanced apoptosis (Figure 18a). NGF‐induced phosphorylation of TrkA and MAPK is diminished in Ndn‐null DRG explants (Figure 18b). These findings indicate that necdin potentiates the NGF signal transduction by enhancing the interaction between these NGF receptors (Figure 18c). NGF induces endosomal association of p75NTR and necdin (Bronfman et al., 2003). This suggests that signaling endosomes containing necdin, p75NTR and TrkA transmit NGF signals from nerve terminals to cell bodies of sensory neurons via the retrograde axonal transport. Necdin also interacts with activated TrkB, a transmembrane receptor of BDNF (brain‐derived neurotrophic factor), and modulates the BDNF‐TrkB‐MAPK signal transduction (Yoshihara, M. & Yoshikawa, K., unpublished data). Accordingly, necdin may promote differentiation of neurotrophin‐dependent neurons by facilitating neurotrophin receptor‐mediated signal transduction.

### 8.3 GnRH neurons

**FIGURE 19:**
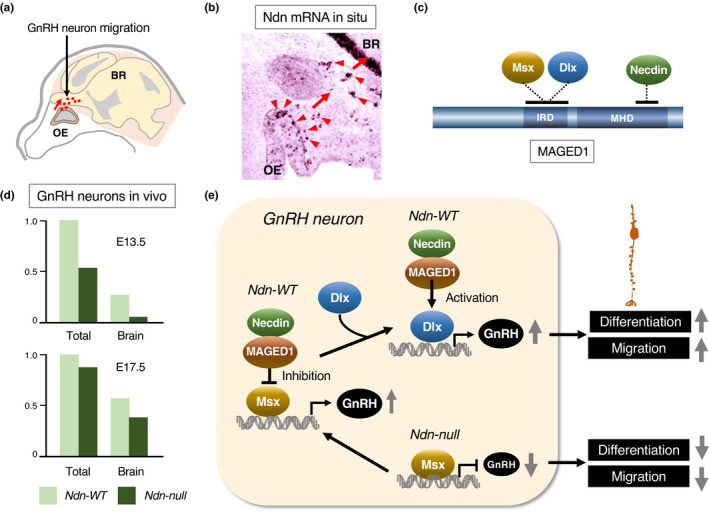
Necdin promotes differentiation of GnRH neurons. (a) Diagram of GnRH neuron migration. GnRH neurons (red dots) migrate from the olfactory epithelium (OE) to the brain (BR) (red arrow) during embryonic period of mouse. (b) In situ hybridization histochemistry for necdin mRNA. Cells with strong necdin mRNA signals (red arrowheads) are presumptive GnRH neurons (Takagi, K. & Yoshikawa, K., unpublished data). (c) A multiprotein complex of necdin, Msx/Dlx and MAGED1. MAGED1 interacts with necdin at the MHD and with Msx/Dlx homeodomain proteins at interspersed hexapeptide repeat domain (IRD). (d) Impaired migration of GnRH neurons. Total GnRH‐immunopositive neurons and those located in the brain were quantified at E13.5 and E17.5. Adapted from Miller et al. (2009). (e) Diagram of necdin‐promoted GnRH neuron differentiation. Msx inhibits GnRH neuron differentiation, and necdin, together with MAGED1, counteracts the inhibitory effect of Msx. The necdin/MAGED1 complex enhances Dlx2‐promoted GnRH neuron differentiation. Illustration based on Kuwajima et al. (2004), Kuwajima et al. (2006) and Miller et al. (2009)

GnRH (gonadotropin‐releasing hormone), which is secreted from a specific group of hypothalamic neurons, stimulates the release of gonadotropins from the anterior pituitary. At early stages of development, these GnRH neurons arise in the olfactory placode and migrate to the hypothalamus (Figure 19a). Necdin mRNA is abundantly expressed in presumptive GnRH neurons (Figure 19b). The homeobox transcription factors Msx (msh homeobox) and Dlx (distal‐less homeobox) antagonistically control the differentiation of GnRH neurons (Miller et al., 2009). Msx/Dlx homeodomain proteins interact directly with MAGED1 (Dlxin‐1) (Masuda et al., 2001). Necdin interacts with Msx/Dlx via MAGED1 to form a ternary complex (Kuwajima et al., 2004, 2006) (Figure 19c). The population of GnRH neurons is significantly reduced in the brain of Ndn‐null mouse embryos owing to abnormal migration of GnRH neurons (Miller et al., 2009) (Figure 19d). Interestingly, necdin relieves Msx‐mediated repression of GnRH neuron differentiation but potentiates the Dlx2‐mediated promotion (Figure 19e). These findings raise the possibility that the absence of necdin causes hypogonadotropic hypogonadism due to the abnormal development of GnRH neurons. Since necdin expression is positively regulated by NHLH transcription factors (see Figure 4), NHLH1/NHLH2 double knockout mice, in which necdin expression is markedly reduced, show a marked reduction in the population of GnRH neurons (Kruger et al., 2004). Moreover, NHLH2‐null mice exhibit hypogonadism and obesity (Good et al., 1997). These phenotypes of NHLH mutants are similar to those of Ndn‐null mice, suggesting that NHLH‐mediated Ndn expression contributes to GnRH neuron differentiation.

### 8.4 GABAergic interneurons

**FIGURE 20:**
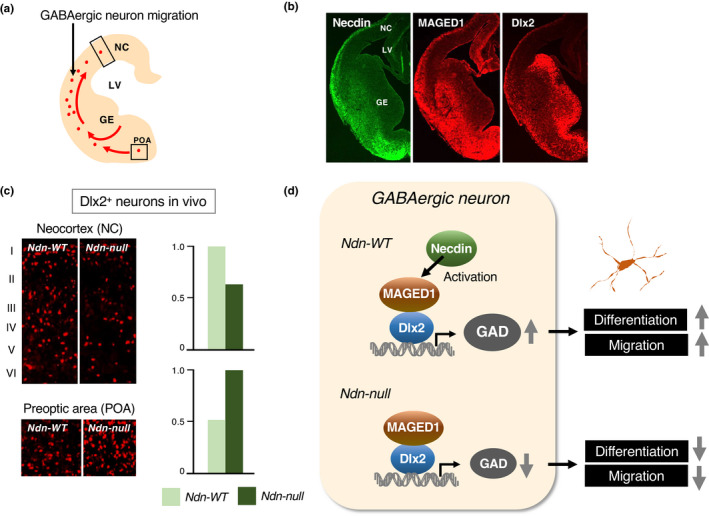
Necdin promotes differentiation of GABAergic interneurons. (a) Diagram of GABAergic neuron migration. GABAergic interneurons in the mouse forebrain arise in the ganglionic eminence (GE) and migrate to the neocortex (NC) during the embryonic period. POA, preoptic area. (b) Expression of necdin, MAGED1 and Dlx2 in the forebrain. Forebrain sections of E13.5 mice were immunohistochemically stained for individual proteins. (c) Dlx2‐expressing neurons in neonatal forebrain. Forebrain sections of P0 mice were immunostained for Dlx2 and analyzed by Dlx2‐immunopositive cell count in NC and POA (Dlx2^+^ neurons). I‐VI, neocortical layers. For experimental details (b, c), see Kuwajima et al. (2006). (d) Diagram of necdin‐promoted GABAergic neuron differentiation. Dlx2 promotes differentiation of GAD‐expressing GABAergic neurons. Necdin promotes the differentiation and migration of GABAergic neurons by interacting with Dlx2 via MAGED1

Cortical inhibitory interneurons project their short axons to excitatory neurons in their vicinity and form local circuits. These neurons, which use GABA (γ‐aminobutyric acid) for synaptic transmission, exhibit highly heterogeneous morphologies (Markram et al., 2004). These neurons arise from NSPC pools in subcortical structures such as ganglionic eminences during early embryonic period and migrate tangentially to cortical regions (Lim et al., 2018) (Figure 20a). Differentiation and specification of GABAergic interneurons in mouse embryonic forebrain are dependent on Dlx homeobox gene family (Anderson et al., 1997). Necdin binds to Dlx2 via MAGED1 to form a ternary complex (Kuwajima et al., 2006). Necdin, MAGED1 and Dlx2 are expressed along the migratory routes of these interneurons (Figure 20b). Over‐expression of necdin in forebrain slices in vitro by electroporation increases the population of neurons expressing GAD (glutamic acid decarboxylase), a GABAergic neuron marker. Ndn‐null mice exhibit a significant reduction in the number of GABAergic neurons both in vivo and in vitro. In Ndn‐null mice at the neonatal stage, the Dlx2‐expressing cell population decreases in the neocortex and increases in the preoptic area, suggesting the abnormal migration of GABAergic interneurons (Kuwajima et al., 2010) (Figure 20c). These data indicate that necdin promotes the differentiation and migration of GABAergic neurons by interacting with Dlx2 via MAGED1 (Figure 20d).

### 8.5 Suppression of astrocyte differentiation

**FIGURE 21:**
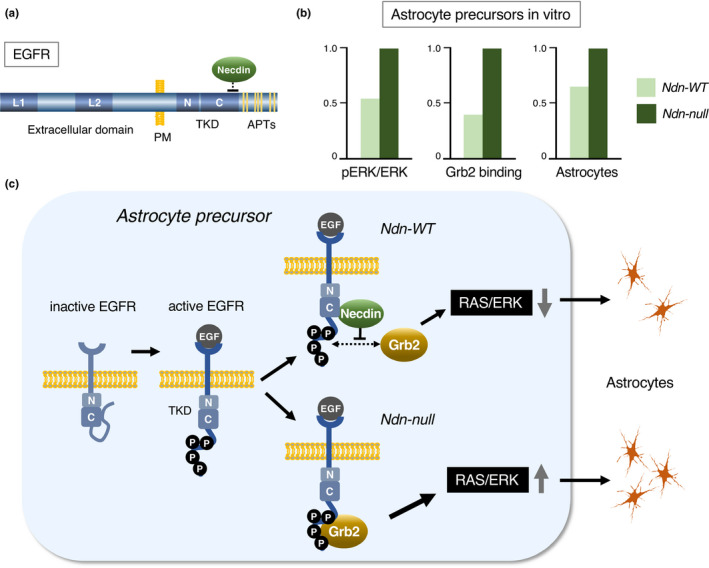
Necdin suppresses astrocyte differentiation. (a) Interaction between necdin and EGF receptor (EGFR). Necdin binds to the C‐lobe of EGFR tyrosine kinase domain (TKD) in an autophosphorylation‐dependent manner. Abbreviations: L1, 2, ligand‐binding domains 1, 2; PM, plasma membrane; N, TKD N‐lobe; C, TKD C‐lobe; and APTs, autophosphorylated tyrosine residues. (b) EGFR‐mediated signal transduction. Astrocyte precursors prepared from the neocortex of E14.5 mice were treated with EGF for 5 min. For EGF signal transduction, cell lysates were immunoblotted for phospho‐ERK and ERK (pERK/ERK). For Grb2 binding, cell lysates were immunoprecipitated with anti‐Grb2 antibody and immunoblotted for EGFR. For astrocyte differentiation, cells were immunostained for the astrocyte marker GFAP. For experimental details (a, b), see Fujimoto et al. (2016). (c) Diagram of necdin‐mediated suppression of astrocyte differentiation. Necdin binds to EGF‐activated EGFR, blocks the interaction between EGFR and Grb2 and suppresses the EGF signal transduction to inhibit astrocyte differentiation

Astrocytes or astroglial cells are non‐neuronal cells enriched in the brain and contribute to the maintenance of normal brain functions such as nutritional support for neurons and repair processes for brain lesions. Astrocytes differentiate from astrocyte precursors (APs) at a late stage (gliogenic phase) of neural stem cell differentiation (Temple, 2001). EGF (epidermal growth factor) and its receptor tyrosine kinase (EGFR) play key roles in controlling proliferation and differentiation of APs. Necdin interacts with the tyrosine kinase domain of autophosphorylated EGFR and represses EGFR‐mediated RAS/ERK signaling pathway (Fujimoto et al., 2016) (Figure 21a). EGF‐induced ERK phosphorylation is enhanced in Ndn‐null APs, in which the interaction between EGFR and the adaptor protein Grb2 is strengthened (Figure 21b), suggesting that necdin suppresses the EGFR/ERK signaling pathway in APs. Consequently, Ndn‐null APs differentiate efficiently into astrocytes. These findings suggest that necdin restrains astrocyte differentiation by suppressing the EGF signaling pathway (Figure 21c). Interestingly, MAGEA1, a type I MAGE member, also interacts with EGFR and, in contrast to necdin, promotes astroglial differentiation, which is counteracted by necdin (Fujimoto et al., 2016). Noteworthily, a gene expression network modeling study reveals that necdin suppresses glioblastoma cell growth via molecular interaction networks involving EGFR (Jornsten et al., 2011). Amplification and mutation of the EGFR gene are often seen in primary glioblastoma cells (Zhu & Parada, 2002). These findings raise the possibility that necdin is involved in the pathogenesis of glioblastoma.

## 9 CELL FUNCTION IV: MAINTENANCE OF ENERGY HOMEOSTASIS

### 9.1 Interaction with Sirt1

**FIGURE 22:**
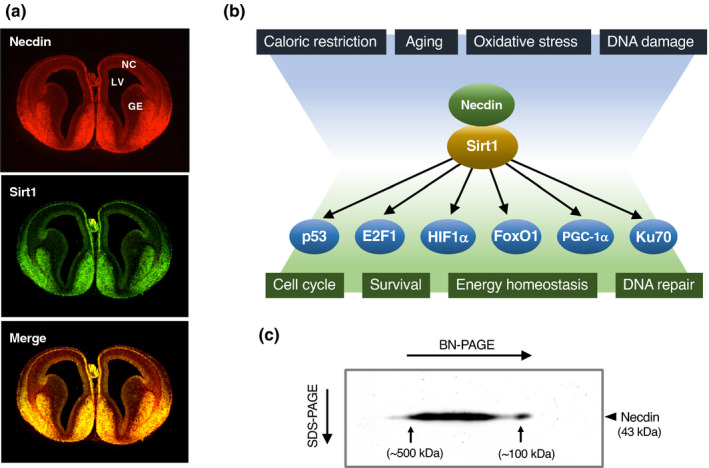
Necdin forms neuronal multiprotein complexes with Sirt1. (a) Distribution of necdin and Sirt1 in mouse forebrain. Forebrain sections prepared from E14.5 mice were double‐immunostained for necdin and Sirt1 for fluorescence microscopy. See Figure 2 for abbreviations (Hasegawa, K. & Yoshikawa, K., unpublished data). (b) Diagram of interactions between necdin, Sirt1 and Sirt1‐binding proteins. Sirt1 has numerous target proteins involved in various cellular functions. Necdin enhances Sirt1‐mediated deacetylation by interacting with Sirt1 and its substrate proteins. Note the overlapped targets of necdin and Sirt1. (c) Necdin‐containing multiprotein complexes in the embryonic brain. Whole brain extract from mouse embryos (E14.5) was analyzed by blue‐native polyacrylamide gel electrophoresis (BN‐PAGE) (4%–20% gradient) and subsequent SDS‐PAGE (10%). Proteins electroblotted onto a PVDF membrane were immunoblotted for necdin. Arrows point the protein sizes ~100 and ~500 kDa (Kashiwagi, H. & Yoshikawa, K., unpublished data)

Sirt1, a mammalian NAD‐dependent protein deacetylase, plays a pivotal role in energy homeostasis, DNA repair and cell survival (Haigis & Sinclair, 2010; McBurney et al., 2013). Sirt1‐null mice mostly die during the early postnatal period and occasionally show abnormal brain development such as exencephaly during the embryonic period (McBurney et al., 2003). Necdin and Sirt1 are strongly expressed in the brain and show similar expression patterns (Figure 22a). Necdin interacts with both Sirt1 and p53 to form a ternary complex and facilitates deacetylation of p53 in neurons (Hasegawa & Yoshikawa, 2008) (see Figure 16). Moreover, several necdin interactors overlap the substrates or interactors of Sirt1. These findings suggest that necdin forms multiprotein complexes with Sirt1 and Sirt1‐binding proteins in neurons (Figure 22b). Embryonic mouse brain contains large‐sized necdin‐immunoreactive complexes ranging from ~100 to ~500 kDa (Figure 22c). This supports the idea that necdin forms neuronal multiprotein complexes with its interactors including Sirt1 and Sirt1‐binding proteins.

Sirt1 mediates calorie restriction‐induced physiological changes leading to health and longevity in mammals (Guarente, 2007). Sirt1 expression is regulated by environmental stimuli such as fasting and exercise, and its enzyme activity is modulated through protein–protein interactions (Haigis & Sinclair, 2010). Noteworthily, Ndn expression is up‐regulated by caloric restriction and down‐regulated by aging in mouse skeletal muscle (Lee et al., 1999). This suggests a close link between necdin and Sirt1 in the control of energy metabolism.

### 9.2 Hypothalamus–pituitary–thyroid axis

**FIGURE 23:**
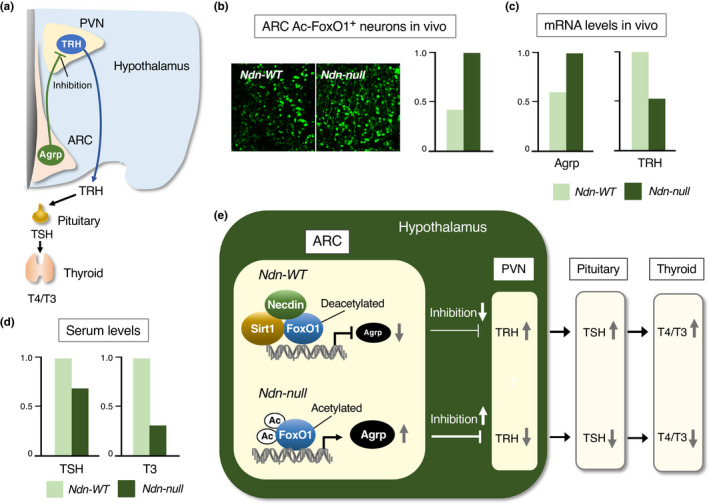
Necdin maintains thyroid function via hypothalamus–pituitary**–**thyroid axis. (a) Diagram of the hypothalamus–pituitary–thyroid axis. Neurons containing Agrp (agouti‐related neuropeptide) in the arcuate nucleus (ARC) send inhibitory axons to TRH (thyrotropin‐releasing hormone)‐producing neurons in the paraventricular nucleus (PVN). TRH promotes TSH (thyroid‐stimulating hormone) secretion from the pituitary, and TSH stimulates T4/T3 (thyroxine/triiodothyronine) secretion from the thyroid. (b) Acetylated FoxO1 expression in ARC. Hypothalamic sections were immunostained for acetylated FoxO1 (Ac‐FoxO1) (images), and the Ac‐FoxO1 intensity was quantified by fluorescence microphotometry (graph). (c) Hypothalamic Agrp and TRH mRNA levels at P30. Hypothalamic mRNA levels were quantified by qRT‐PCR. (d) Serum levels of TSH and T3 at P30. Serum hormone levels were measured by enzyme‐linked immunosorbent assay. For experimental details (b‐d), see Hasegawa et al. (2012). (e) Diagram of necdin‐promoted thyroid function. Necdin promotes Sirt1‐mediated FoxO1deacetylation, suppresses Agrp expression in ARC, activates TRH neurons in PVN and increases serum levels of TSH and T4/T3

Thyroid hormones play important roles in the energy homeostasis and brain development (Anderson, 2001). Production of thyroid hormones and maintenance of their serum levels are strictly controlled via the hypothalamus–pituitary–thyroid axis (Figure 23a). In the hypothalamus, GABAergic inhibitory neurons expressing Agrp in the ARC project their axons to TRH‐producing neurons in the paraventricular nucleus. FoxO1, a forkhead transcription factor mediating insulin signals, up‐regulates Agrp expression in the hypothalamus (Kitamura et al., 2006). Necdin forms a ternary complex with Sirt1 and FoxO1, diminishes FoxO1 acetylation and suppresses FoxO1‐mediated Agrp gene transactivation (Hasegawa et al., 2012). Ndn‐null mice express high levels of acetylated FoxO1 in vivo in the ARC (Figure 23b), indicating that necdin down‐regulates FoxO1 acetylation. In Ndn‐null mice, hypothalamic Agrp mRNA levels increase, and TRH mRNA levels decrease (Figure 23c). Serum levels of TSH and thyroid hormones also decrease significantly (Figure 23d). Collectively, necdin regulates the acetylation levels of FoxO1 in hypothalamic neurons to maintain the thyroid function via the hypothalamus–pituitary–thyroid axis (Figure 23e).

### 9.3 Mitochondrial biogenesis

**FIGURE 24:**
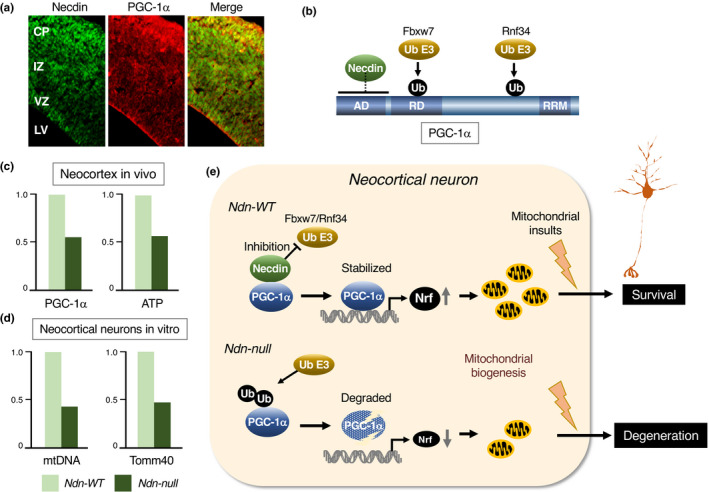
Necdin promotes mitochondrial biogenesis in neocortical neurons. (a) Expression of necdin and PGC‐1α in the neocortex. Neocortical sections of E14.5 mouse forebrain were double‐immunostained for necdin and PGC‐1α. Note the high expression levels of necdin and PGC‐1α in the cortical plate (CP). See Figure 2 for abbreviations. (b) Domain structure of PGC‐1α. Necdin binds to the activation domain (AD) of PGC‐1α. Abbreviations: RD, repression domain; RRM, RNA recognition motif; Ub E3, ubiquitin E3 ligases (Fbxw7, Rnf34); and Ub, ubiquitin. (c) Neocortical PGC‐1α and ATP levels in vivo. Neocortical levels of PGC‐1α and ATP from E14.5 mice were quantified by immunoblot/densitometry and chemiluminometry, respectively. (d) Mitochondrial DNA and Tomm40 levels in neocortical neurons. Mitochondrial D‐loop DNA (mtDNA) and Tomm40 mRNA levels in primary neocortical neurons were analyzed by quantitative PCR and qRT‐PCR, respectively. For experimental details (a‐d) see Hasegawa et al. (2016). (e) Diagram of necdin‐promoted mitochondrial biogenesis in neocortical neurons. Necdin binds directly to PGC‐1α and prevents E3 ubiquitin ligase (Ub E3)‐mediated ubiquitination of PGC‐1α. Stabilized PGC‐1α transactivates mitochondrial biogenesis‐promoting genes such as Nrf. Necdin‐expressing neurons are resistant to mitochondrial insults

Neurons require large amounts of ATP generated by neuronal mitochondria for their electric activities and synaptic transmission (Attwell & Laughlin, 2001; Bailey, 2019). Moreover, neurons heavily rely on mitochondria for their survival (Nicholls & Budd, 2000). Mitochondrial dysfunction leads to pathological aging of neurons and neurodegenerative diseases (Yankner et al., 2008). Thus, the biogenesis and function of neuronal mitochondria must be properly maintained for normal brain activities. PGC‐1α (peroxisome proliferator‐activated receptor γ coactivator‐1α) is a master regulator that orchestrates gene expression required for mitochondrial biogenesis and function (Scarpulla et al., 2012). Necdin and PGC‐1α are strongly expressed in neocortical neurons (Hasegawa et al., 2016) (Figure 24a). Necdin expression levels correlate well with PGC‐1α levels in the brain during development and aging. Necdin binds to PGC‐1α and prevents ubiquitin‐dependent degradation of PGC‐1α (Figure 24b). Neocortical levels of PGC‐1α and ATP are significantly reduced in Ndn‐null mice (Figure 24c). Moreover, expression levels of the mitochondria‐specific genes mitochondrial DNA and Tomm40 are markedly reduced in primary neocortical neurons prepared from Ndn‐null mice (Figure 24d). Ndn‐null neurons are highly susceptible to mitochondrial toxins such as mitochondria‐specific enzyme inhibitors. These data indicate that necdin promotes neuronal mitochondrial biogenesis and function by stabilizing PGC‐1α to protect neurons against mitochondrial insults (Figure 24e).

## 10 PROTEIN–PROTEIN INTERACTION

### 10.1 Interacting proteins

Necdin interacts with many proteins located in different subcellular compartments (Table 2). Interactions with these proteins are analyzed mostly by biochemical assays such as co‐immunoprecipitation, two‐hybrid assay and pull‐down assay, which are adopted for detecting permanent protein–protein interactions (PPIs) (Perkins et al., 2010). Most of the interactions between necdin and listed proteins are confirmed by functional assays. Although a number of necdin interactors have been identified by large‐scale screening methods such as Ras recruitment system (Lavi‐Itzkovitz et al., 2012) and affinity captured purification/mass spectroscopy (Sanderson et al., 2020), only the proteins whose interactions with necdin are confirmed by other measures are listed here. The necdin‐interacting proteins are expediently grouped as below.

**TABLE 2:** Necdin interactors

Protein (alias)	Description	Reference
Oncovirus		
SV40 Large T antigen	Simian virus 40 oncoprotein inducing cellular transformation	Taniura et al. (1998)
Adenovirus E1A	Adenovirus oncoprotein inducing cellular transformation	Taniura et al. (1998)
Transcriptional regulation		
E2F1	E2F transcription factor family regulating the cell cycle	Taniura et al. (1998)
E2F4	E2F transcription factor family regulating cell differentiation	Kobayashi et al. (2002)
p53 (TP53)	Transcription factor with tumor suppressor activity, inducing apoptosis	Taniura et al. (1999)
HIF1α	Hypoxia‐Inducible factor 1α, bHLH‐PAS transcription factor	Moon et al. (2005)
HIF2α (EPAS)	Hypoxia‐Inducible factor 2α, bHLH‐PAS transcription factor	Huang et al. (2013)
ARNT2	Aryl hydrocarbon receptor nuclear translocator 2, bHLH‐PAS transcription factor	Friedman and Fan (2007)
BMAL1(ARNTL)	bHLH‐PAS transcription factor controlling circadian rhythm generation	Lu et al. (2020)
Msx1/2	Msh homeobox 1/2‐encoded protein, muscle differentiation suppressor interacting with MAGED1	Kuwajima et al. (2004)
Dlx2/5	Distal‐less homeobox 2/5‐encoded protein, neuron differentiation promoter interacting with MAGED1	Kuwajima et al. (2006)
Bmi1 (PCGF4, RNF51)	Polycomb group RING finger protein promoting proliferation of neural stem cells	Minamide et al. (2014)
FoxO1 (FKHR)	Forkhead transcription factor regulating energy homeostasis	Hasegawa et al. (2012)
PGC‐1α (PPARGC1A)	Peroxisome proliferator‐activated receptor γ coactivator promoting mitochondrial biogenesis	Hasegawa et al. (2016)
Signal transduction		
p75NTR (NGFR)	Neurotrophin receptor mediating apoptosis and survival signals	Tcherpakov et al. (2002)
TrkA (NTKR1)	Tropomyosin‐related kinase (Trk) A mediating NGF signal for differentiation	Kuwako et al. (2005)
EGFR (ErbB‐1)	Epidermal growth factor receptor tyrosine kinase promoting cell proliferation	Fujimoto et al. (2016)
GNAO1(Gαo)	Guanine nucleotide‐binding protein Go subunit a promoting neuronal differentiation	Ju et al. (2014)
Pre‐interleukin‐1α	Interleukin‐1α precursor mediating cell migration and differentiation	Hu et al. (2003)
EID‐1	EP300‐interacting inhibitor of differentiation‐1 suppressing myogenic differentiation	Bush and Wevrick (2008)
NME1 (Nm23‐H1)	Tumor metastasis suppressor, cellular target of Epstein–Barr virus oncoprotein EBNA3C	Kaul et al. (2009)
CCAR1 (CARP1)	Cell division cycle and apoptosis regulator protein‐1 mediating apoptosis signals	Francois et al. (2012)
Nucleobindin 2 (NEFA)	Calcium‐binding Leu‐zipper protein, precursor of nesfatin (satiety‐promoting peptide)	Taniguchi et al. (2000)
Leptin receptor (LEPR)	Leptin receptor mediating satiety signals for energy balance control	Wijesuriya et al. (2017)
Cellular compartmentation		
hnRNP U (SAF‐A)	Heterogeneous nuclear ribonucleoprotein U, nuclear matrix‐associated RNA/DNA‐binding protein	Taniura and Yoshikawa (2002)
PAIP2	Polyadenylate (polyA)‐binding protein‐interacting protein	Sanderson et al. (2020)
Fez1/2	Fasciculation and elongation protein ζ‐1/2 implicated in axonal outgrowth	Lee et al. (2005)
BBS4	Bardet–Biedl syndrome 4 implicated in ciliogenesis and centrosome formation	Lee et al. (2005)
Nogo‐A (Reticulon 4)	Neurite outgrowth inhibitor suppressing axonal regeneration	Liu et al. (2009)
Dysbindin	Dystrobrevin binding protein 1 present in axon bundles and terminals	Ma et al. (2011)
Transportin 1 (Importin‐β 2)	Karyopherin involved in nucleocytoplasmic transport	Lavi‐Itzkovitz et al. (2012)
SUGT1(SGT1)	Kinetochore complex assembly cochaperone interacting with heat shock protein 90	Lu et al. (2020)
Protein modification		
Sirt1	NAD‐dependent histone deacetylase sirtuin 1 regulating energy homeostasis	Hasegawa and Yoshikawa (2008)
PIAS1	Protein inhibitor of activated STAT, SUMO E3 ligase	Gur et al. (2014)
RanBP2 (Nup358)	RAN‐binding protein 2, a nuclear pore component possessing E3 SUMO‐protein ligase activity	Fujiwara et al. (2016)
Senp2	Sentrin‐specific protease 2, SUMO deconjugating enzyme associated with nuclear pore complex	Fujiwara et al. (2016)
Dimerization		
MAGED1 (NRAGE, Dlxin‐1)	Type II MAGE protein expressed abundantly in neuron and skeletal muscle	Kuwajima et al. (2004)
MAGEL2	Type II MAGE protein encoded in paternally expressed gene	Wijesuriya et al. (2017)
Necdin	Homodimerization	Tcherpakov et al. (2002)

#### 10.1.1 Transcriptional regulation

Necdin binds to Rb‐binding domains of E2F1 and E2F4 and regulates their functions (see Figure 11) (Kobayashi et al., 2002; Taniura et al., 1998). Necdin interacts with p53, suppresses p53‐dependent transactivation and inhibits p53‐mediated apoptosis (see Figure 15) (Taniura et al., 1999). In addition to these major transcription factors, necdin interacts with bHLH‐PAS (basic helix‐loop‐helix with Per‐Arnt‐Sim domain) family transcription factors. Necdin binds to the ODD (oxygen‐dependent degradation) domain of HIF‐1α and promotes the degradation of HIF‐1α under hypoxic conditions (Moon et al., 2005). HIF‐2α interacts with necdin via its PAS domain and promotes hypoxia‐induced degradation of necdin in NSPCs (see Figure 13) (Huang et al., 2013). Necdin binds to ARNT2, a dimer partner of SIM1 (single‐minded homolog 1), and represses ARNT2‐SIM1 complex‐mediated transcriptional activation (Friedman & Fan, 2007). Necdin also interacts with BMAL1, a key bHLH‐PAS transcription factor responsible for circadian rhythm generation, and stabilizes BMAL1 (Lu et al., 2020). Necdin interacts indirectly with Msx/Dlx homeodomain proteins via MAGED1 and modulates the effects of Msx/Dlx on differentiation of specific neurons and skeletal muscle (Kuwajima et al., 2004, 2006). Necdin binds to Bmi1, a polycomb group complex protein promoting stem cell proliferation, and relieves Bmi1‐dependent suppression of p16 expression (see Figure 12), whereas Bmi1 relieves necdin‐mediated suppression of E2F1‐dependent Cdk1 expression (Minamide et al., 2014). Necdin interacts with FoxO1, enhances Sirt1‐mediated FoxO1 deacetylation and suppresses FoxO1‐dependent transactivation (see Figure 23) (Hasegawa et al., 2012). Necdin binds to PGC‐1α, stabilizes PGC‐1α and promotes PGC‐1α‐mediated mitochondrial biogenesis in postmitotic neurons (see Figure 24) (Hasegawa et al., 2016).

#### 10.1.2 Signal transduction

Necdin binds to p75NTR and accelerates neurite outgrowth in response to NGF (Tcherpakov et al., 2002). Necdin interacts with TrkA, a p75NTR‐interacting partner, promotes association between TrkA and p75NTR and enhances NGF signal transduction (see Figure 18) (Kuwako et al., 2005). Necdin binds to EGFR and suppresses EGFR‐mediated signal transduction for astrocyte differentiation (see Figure 21) (Fujimoto et al., 2016). Activated GNAO1 (Gαo) binds to necdin and augments necdin‐mediated cellular responses such as mitotic suppression and neurite outgrowth (Ju et al., 2014). Pre‐interleukin‐1α, an unprocessed interleukin‐1α precursor implicated in systemic sclerosis, interacts with necdin in the nucleus and counteracts necdin‐mediated suppression of cell growth and collagen expression (Hu et al., 2003). Necdin interacts with EID‐1, a member of Nse4/EID family, relieves EID1‐mediated suppression of myogenesis and promotes myoblast differentiation (Bush & Wevrick, 2008). Furthermore, necdin interacts with all Nse4/EID family members (NSE4a, NSE4b, EID1, EID2 and EID2B) (Hudson et al., 2011). NME1 (Nm23‐H1), a cellular target of the Epstein–Barr virus nuclear antigen EBNA3C, interacts with necdin and antagonizes necdin‐mediated growth suppression and anti‐angiogenic function in cancer cells (Kaul et al., 2009). Necdin binds to CCAR1, a regulator of cell division and apoptosis, facilitates degradation of CCAR1 and enhances myoblast survival (Francois et al., 2012). Necdin interacts with nucleobindin 2 (NEFA), a precursor of hypothalamic satiety‐promoting neuropeptide nesfatin‐1 (Taniguchi et al., 2000). Necdin interacts with the leptin receptor (LEPR) and, together with MAGEL2, controls LEPR sorting and degradation (Wijesuriya et al., 2017).

#### 10.1.3 Cellular compartmentation

Necdin and hnRNP U, a nuclear matrix‐binding protein, interact with each other and cooperatively suppress mitosis (Taniura & Yoshikawa, 2002). Necdin binds to PAIP2, a poly(A)‐binding protein‐interacting protein, and increases PAIP2 stability (Sanderson et al., 2020). Necdin interacts with Fez1/2, fasciculation and elongation proteins implicated in axonal outgrowth, and prevents proteasomal degradation of Fez1 (Lee et al., 2005). Necdin binds to BBS4, a protein implicated in the Bardet–Biedl syndrome, and forms a BBS4‐containing multiprotein complex at the centrosomes (Lee et al., 2005). Nogo‐A, an inhibitor of axonal regeneration, binds to necdin and inhibits necdin‐mediated neurite outgrowth (Liu, Wang, et al., 2009). Dysbindin‐1 interacts with necdin and attenuates necdin‐mediated repression of p53‐dependent transactivation (Ma et al., 2011). Transportin1 binds to necdin, translocates necdin from the nucleus and induces extensive cell death (Lavi‐Itzkovitz et al., 2012). Necdin binds to SGT1 and enables SGT1‐HSP90 (heat shock protein 90) chaperone machinery to stabilize BMAL1 (Lu et al., 2020).

#### 10.1.4 Protein modifications

Necdin binds to Sirt1 and promotes Sirt1‐mediated deacetylation of Sirt1 substrates such as p53 and FoxO1 (see Figures 16, 21, 23) (Hasegawa et al., 2012; Hasegawa & Yoshikawa, 2008). Necdin binds to PIAS1 SUMO E3 ligase and promotes the degradation of PIAS1 via the ubiquitin–proteasome pathway (Gur et al., 2014). Necdin interacts with RanBP2, a nuclear pore‐associated SUMO E3 ligase, whereas necdin binds to Senp2, a SUMO deconjugating enzyme, which promotes desumoylation of SUMO‐conjugated RanGAP (Fujiwara et al., 2016).

#### 10.1.5 Dimerization

MAGED1 binds to necdin and forms a multiprotein complex containing necdin and Msx/Dlx homeodomain proteins (Kuwajima et al., 2004). MAGEL2 binds to necdin and controls degradation of the leptin receptor (Wijesuriya et al., 2017). Necdin interacts with itself (Tcherpakov et al., 2002), suggesting that necdin forms a homodimer in neurons. Thus, type II MAGE proteins seem to interact with each other via their MAGE homology domains.

### 10.2 Comparison of necdin and NSMCE3

The two NDN subfamily members necdin and NSMCE3 (NDNL2, MAGEG1) share functional similarities in anti‐mitotic and anti‐apoptotic properties (Kuwako et al., 2004). To gain insight into their functional conservation and divergence, their protein‐binding properties are compared (Table 3). Like necdin, NSMCE3 interacts with E2F1 and p75NTR, though their binding domains of the p75NTR cytoplasmic region differ (Kuwako et al., 2004). These MAGE proteins are likely to shuttle between the plasma membrane and the nucleus depending on their targets p75NTR and E2F1. Noteworthily, chicken MAGE (NSMCE3) also interacts with E2F1 and p75NTR (Lopez‐Sanchez et al., 2007). In contrast, necdin and NSMCE3 interact with SUMO E3 ligases in a different manner. Necdin but not NSMCE3 binds to PIAS (protein inhibitor of activated STAT) SUMO E3 ligase (Gur et al., 2014). Although the type I MAGE protein MAGEA1 interacts with the SUMO E3 ligases NSMCE2 and Cbx4, neither necdin nor NSMCE3 binds to these proteins (Gur et al., 2014). MAGE family proteins also interact with E3 RING ubiquitin ligases (Lee & Potts, 2017; Weon & Potts, 2015). NSMCE3 but not necdin interacts with NSMCE1, the SMC5/6 subcomplex element with ubiquitin E3 ligase activity (Doyle et al., 2010). This may account for the presence of NSMCE3 in the SMC5/6 complex of human HeLa cells (Taylor et al., 2008) and the absence of necdin in that of mouse neural stem cells (Katayama, Y. & Yoshikawa, K., unpublished data) (see Figure 8). Neither necdin nor NSMCE3 binds to TRIM27, another E3 RING ubiquitin ligase that interacts with MAGEL2 and MAGEF1 (Doyle et al., 2010). Necdin and NSMCE3 interact differently with NSMCE4/EID family proteins. Necdin binds to all NSMCE4/EID family members (NSMCE4a, NSMCE4b, EID1, EID2, EID2b), whereas NSMCE3 binds only to NSMCE4a and NSMCE4b (Hudson et al., 2011). Interestingly, MAGEF1 binds to EID1, EID2 and EID2b, but to neither NSMCE4a nor NSMCE4b (Hudson et al., 2011). These binding protein selectivities of MAGE proteins provide useful information about the functional conservation and diversification of mammalian MAGE proteins.

**TABLE 3:** Protein binding properties of necdin and NSMCE3

Interactor	Necdin	NSMCE3	Reference
E2F1	+	+	Kuwako et al. (2004)
p75NTR	+	+	Kuwako et al. (2004)
PIAS1	+	−	Gur et al. (2014)
Cbx4	−	−	Gur et al. (2014)
NSMCE2	−	−	Gur et al. (2014)
NSMCE1	−	+	Doyle et al. (2010)
NSMCE4a	+	+	Hudson et al. (2011)
NSMCE4b	+	+	Hudson et al. (2011)
EID1	+	−	Hudson et al. (2011)
EID2	+	−	Hudson et al. (2011)
EID2b	+	−	Hudson et al. (2011)

### 10.3 Protein interaction networks

**FIGURE 25:**
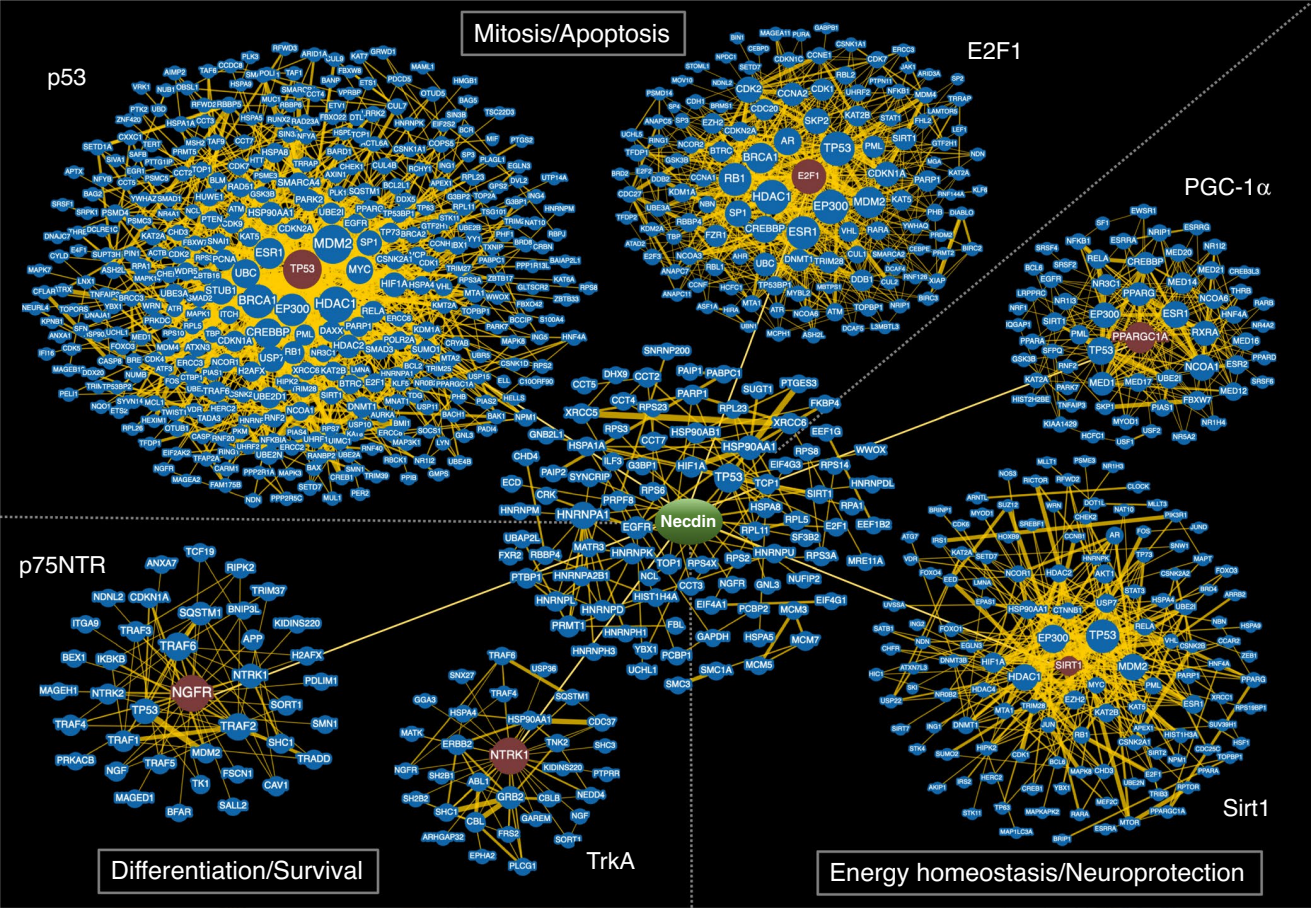
Necdin targets major hub proteins of protein–protein interaction networks. Necdin interacts with major hub proteins such as p53 (TP53), E2F1, Sirt1, PGC‐1α (PPARGC1A), TrkA (NTRK1) and p75NTR (NGFR). Networks with these hubs are classified expediently into functional modules of mitosis/apoptosis, differentiation/survival and energy homeostasis/neuroprotection. The node size reflects the number of attached edges. The number of nodes in each network is limited at the minimum evidence (the number of curated interactions): 2 for E2F1, PGC‐1α, TrkA and p75NTR; and 5 for p53, Sirt1 and necdin. Illustration based on the data from Biological General Repository for Interaction Datasets (BioGRID https://thebiogrid.org/)

As necdin interacts with many proteins, necdin is most likely to form multiprotein complexes in a context‐dependent manner during neuronal development. Necdin‐interacting proteins such as p53, E2F1, p75NTR, TrkA, Sirt1 and PGC‐1α serve as major hubs (i.e., nodes with high density of links) of PPI networks (Figure 25). These hub proteins contribute to the regulation of gene expression, cell growth and differentiation and form modular architectures: p53 and E2F1 in mitosis/apoptosis; p75NTR and TrkA in differentiation/survival; and Sirt1 and PGC‐1α in neuronal energy homeostasis/neuroprotection. Although necdin interacts individually with hub proteins in distinct functional modules, its effects are functionally purposive: The interactions with p53 and E2F1 are suppressive, whereas the interactions with Sirt1, PGC‐1α and neurotrophin receptors (p75NTR/TrkA) are promotive. By linking with these major hubs, necdin may have created its own PPI network for neuronal vitality.

A large number of necdin interactors have been identified using PPI‐based screening strategies. A large‐scale screening using the Ras recruitment system for necdin‐binding proteins in mouse embryonal head reveals a wide range of potential interactors with different subcellular localizations (Lavi‐Itzkovitz et al., 2012). These proteins form modular networks for cytoplasmic protein interactions, nuclear transcription and nucleocytoplasmic transport, indicating the multiple roles of necdin in different subcellular compartments. Another large‐scale PPI‐based screening for necdin interactors in HEK293 cells using protein affinity capture/mass spectrometry yields a number of necdin‐proximate proteins that cluster into modular networks for RNA metabolism and mRNA splicing (Sanderson et al., 2020). Consequently, the PPI network‐based functional analyses of necdin‐binding proteins in different types of cells or tissues will provide information about novel biological roles of necdin.

PPI networks in mammalian cells show typical scale‐free characteristics (Barabási & Oltvai, 2004). The scale‐free network is expanded efficiently by creating new links to large hubs. Necdin interacts with highly connected hub proteins such as p53 and Sirt1. These hubs are evolutionarily old and thus have a better chance to gain many links during the course of evolution (Huart & Hupp, 2013; McBurney et al., 2013). Moreover, necdin per se is a highly connected hub. Therefore, necdin may have exploited the preexisting PPI networks in mammalian ancestors to integrate them for mammalian neuron‐specific functions.

## 11 PROTEIN STRUCTURE

### 11.1 Ordered and disordered domains

**FIGURE 26:**
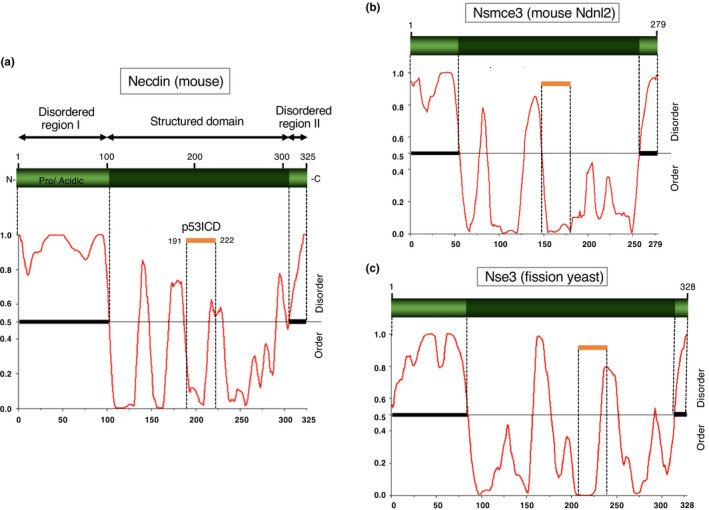
Structurally ordered and disordered domains of necdin and related proteins. Ordered and disordered regions were analyzed by the *predictor of natural disordered regions* (PONDR, http://www.pondr.com/). The ordered (dark green) and disordered regions (light green) of mouse necdin (a), mouse Nsmce3 (Ndnl2) (b), and fission yeast (S. pombe) Nse3 (c). Horizontal orange bars, necdin p53‐interacting core domain (p53ICD) (a) and its homologous regions (b, c)

Computational predictions of natural disorder regions (Dunker et al., 2002) have demonstrated that necdin consists of a large ordered (structured) region and disordered regions at the N‐ and C‐terminus (Figure 26a). The ordered region of necdin (aa 100–310) includes the MAGE homology domain (aa 109–277), and the N‐terminal disordered region corresponds to the proline‐rich acidic region. Mouse Nsmce3 (Ndnl2, Mageg1) (Figure 26b) and yeast Nse3 (Figure 26c) are also composed of large ordered regions and N‐terminal disordered regions. Necdin‐binding proteins such as SV40 large T antigen, E2F1, p53 and nucleobindin 2 interact with the structured domain (Taniguchi et al., 2000; Taniura et al., 1998, 1999). The region aa 191–222 at the center of the structured domain is responsible for p53 interaction, mitotic inhibition and nuclear matrix localization (p53‐interacting core domain, p53ICD) (Taniura et al., 2005). Furthermore, the p53ICD‐corresponding sequences of nonmammalian MAGE (NSMCE3) and yeast Nse3 are highly conserved (Hudson et al., 2011), suggesting the functional importance of this domain.

### 11.2 Homology modeling

**FIGURE 27:**
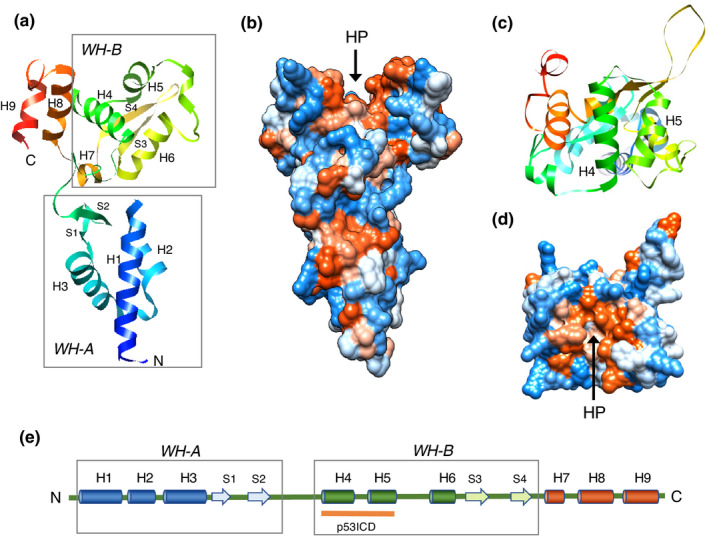
Homology modeling of necdin tertiary structure. (a‐d) Homology modeling of mouse necdin. A tertiary structure of mouse necdin was simulated by template homology‐modeling program (Spanner; https://sysimm.org/spanner/) (Shirai et al., 2014) using the structural data of human NDNL2 (MAGEG1, NSMCE3) (PDB 5WY5) (Doyle et al., 2010). (a, c) ribbon representation; (b, d) surface hydrophobicity representation with hydrophobic (red) and hydrophilic (blue), and neutral (white) aa; (a, b) side view; (c, d) top view. Note the tandem winged‐helix motif (WH‐A/WH‐B) exhibiting a Jomon pottery‐shaped configuration with the hydrophobic pocket (HP, arrows) on the top. (e) Diagram of necdin secondary structure. The necdin structured domain consists of nine helices (H1‐9) and four sheets (S1‐4), including two WH motifs (WH‐A, H1‐3/S1‐2; WH‐B, H4‐6/S3‐4). Orange bar, p53ICD

A tertiary protein structure of necdin is simulated on the basis of the structural data of NDNL2 (MAGEG1, NSMCE3) (PDB 5WY5) (Doyle et al., 2010) using a hybrid template homology‐modeling program (Shirai et al., 2014). The structured domain of necdin consists of two winged‐helix (WH) folds (WH‐A and WH‐B) (Figure 27). The WH fold is a specific helix‐turn‐helix motif that typically comprises three α‐helices (H) and three β‐strands (S) in the order H1‐S1‐H2‐H3‐S2‐S3. The structured domain of necdin consists of H1‐3/S1‐2 (WH‐A) and H4‐6/S3‐4 (WH‐B). Necdin, like NDNL2, has three extra helices formed by the extreme C‐terminal residues (H7‐H9). The WH fold is often seen in nuclear factors having many target molecules and participates in establishing protein–DNA and protein–protein interactions (Teichmann et al., 2012). The fact that WH proteins frequently exhibit exposed patches of hydrophobic residues (Gajiwala & Burley, 2000) indicates that necdin interacts with multiple proteins via its surface hydrophobic regions. As surface hydrophobic regions are important for protein–protein interactions, the topological alignment of hydrophobic aa residues in these regions must be highly conserved in MAGE family members.

### 11.3 p53‐interacting pocket

**FIGURE 28:**
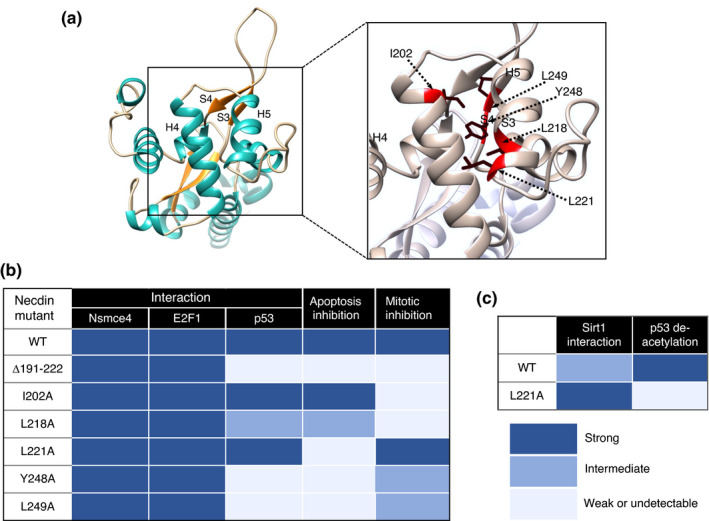
Mutational analysis of p53‐interacting pocket. (a) Tertiary structure of p53‐interacting hydrophobic pocket. The hydrophobic pocket is formed at the WH‐B region of simulated necdin structure (top view). The arrows with broken lines point to mutated hydrophobic aa residues (red) and their side chains (black). (b) Functional analyses of p53‐interacting pocket using necdin mutants. Interactions of necdin mutants were analyzed by co‐immunoprecipitation assay. Cell lysates of HEK293 cells transfected with cDNAs for necdin mutants and necdin interactors (Nsemce4, E2F1, p53) were immunoprecipitated with anti‐necdin antibody NC243 and immunoblotted with antibodies to the necdin interactors. Proteins were quantified by immunoblot/densitometry. For p53‐dependent apoptosis assay (apoptosis inhibition), U2OS cells were transfected with cDNAs for p53 and necdin mutants, and p53‐immunopositive viable cells were counted 72 hr later. For mitotic suppression assay (Mitotic inhibition), HEK293 cells were transfected with cDNAs for necdin mutants and LacZ, incubated for 24 hr, treated with 5‐ethynyl‐2’‐deoxyuridine (EdU) for 3 hr and analyzed for EdU/LacZ‐double positive S‐phase cell count. (c) Effect of L221A mutant on Sirt1‐mediated p53 deacetylation. Interaction between L221A and Sirt1 (Sir1 interaction) was analyzed by immunoprecipitation assay as above. For p53 deacetylation assay (p53 deacetylation), HEK293A cells were transfected with cDNAs for L221A and 6xMyc tagged‐p53. Cell lysates were immunoprecipitated with anti‐Myc antibody and immunoblotted with anti‐acetyl‐K373 p53 antibody for quantification (Tanigawa, S. & Yoshikawa, K., unpublished data)

A hydrophobic pocket structure comprising two helices H4‐H5 and two sheets S3‐S4 is found on top of the simulated necdin tertiary structure (Figure 28a). The p53‐interacting core domain corresponds to this hydrophobic pocket in the second winged‐helix fold (WH‐B). The hydrophobic aa I202, L218, L221, Y248 and L249 in the pocket region are highly conserved within MAGE family proteins (Hudson et al., 2011). These aa residues are substituted with alanine to determine the specific aa responsible for interaction with p53, inhibition of p53‐dependent apoptosis and mitotic inhibition (Figure 28b). All these activities are reduced in necdin Δ191–222 mutant, indicating that the aa 191–222 region forms a functional pocket structure. The mutants I202A and L218A interact with p53 and inhibit p53‐dependent apoptosis but show weak mitotic inhibition, indicating that aa I202 and L218 located at the upper portion of the pocket are critical for mitotic inhibition. In contrast, the mutants Y248A and L249A show reduced p53 interaction but retain mitotic inhibition activity, indicating that aa L248 and L249 at the bottom portion are responsible for p53 interaction. Interestingly, the L221A mutant interacts strongly with both p53 and Sirt1 and strengthens their interaction, but its activities of p53 deacetylation and inhibition of p53‐dependent apoptosis are very weak (Figure 28c). This indicates that aa L221 modulates the strength of Sirt1‐p53 interaction relevant to Sirt1 catalytic activity. Neither Nsmce4 nor E2F1 interacts with these aa substitution mutants. These data suggest that this pocket structure is indispensable for the interaction with p53 and necdin‐mediated mitotic arrest.

## 12 CONCLUSIONS AND PERSPECTIVES

### 12.1 Implications of developmental neurobiology

**FIGURE 29:**
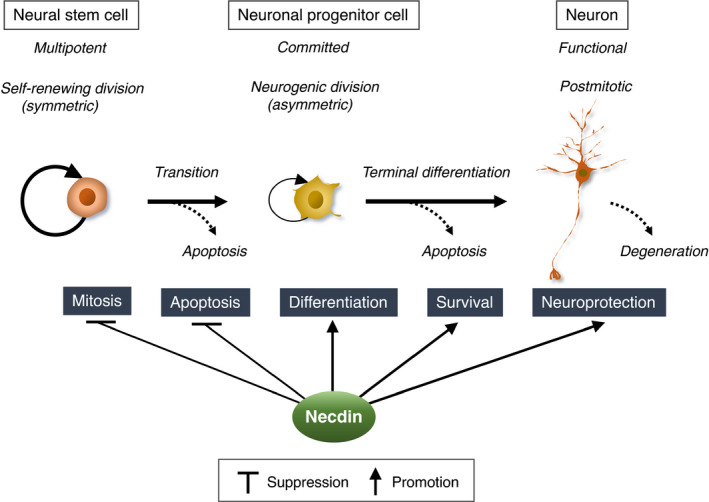
Necdin promotes neuronal vitality throughout the lifetime. Necdin suppresses both mitosis and apoptosis of neural stem cells and neuronal progenitor cells. Necdin also promotes differentiation, suppresses apoptosis, promotes survival and protects postmitotic neurons from detrimental stresses under pathological conditions

Multiple lines of evidence have shown that necdin promotes the vitality of neurons throughout the lifetime (Figure 29). Necdin suppresses proliferation of neural stem cells by interacting with E2F1 and Bmi1, both of which promote proliferation of neural stem cells (see Figure 12). Necdin expression is transcriptionally controlled by major transcription factors that mediate cellular signals for cell fate decisions (see Figure 4). The necdin level is regulated post‐translationally in an oxygen tension‐dependent manner through ubiquitin–proteasome pathway (see Figure 13). Therefore, necdin may act as a molecular rheostat that controls the proliferation of NSPCs in response to cellular contexts and environmental stimuli. Necdin deficiency enhances the proliferation of NSPCs, whereas necdin concomitantly promotes apoptosis of hyperproliferative NSPCs (see Figure 12). These findings suggest that necdin prevents NSPCs from uncontrolled mitosis that potentially induces genomic instability leading to p53‐mediated apoptosis during neuronal development.

Necdin promotes differentiation of specific neurons by interacting with regulatory proteins involved in neuronal specification. In sensory neurons, necdin interacts with neurotrophin receptors involved in cell fate decisions (see Figure 18). In the absence of necdin, subpopulations of NSPCs fail to differentiate properly and undergo apoptosis of neurotrophin‐dependent neurons. Necdin also promotes differentiation of hypothalamic GnRH neurons and neocortical GABAergic neurons by interacting with Dlx/Msx homeodomain proteins (see Figures 19, 20). The abnormal migration of GnRH neurons and GABAergic interneurons may lead to defective neuronal network formation and consequent functional abnormalities.

Several lines of evidence suggest that unscheduled cell cycle entry or aberrant mitosis of postmitotic neurons causes neurodegeneration (Klein & Ackerman, 2003). Cell cycle regulators such as E2F1, Rb and cyclin‐dependent kinases such as Cdk1 are expressed in postmitotic neurons and potentially induce aberrant reentry into the cell cycle under pathological conditions (Joseph et al., 2020; Yoshikawa, 2000). Genotoxic compounds induce apoptosis accompanied by cell cycle reentry of postmitotic neurons, suggesting that cell cycle activation is a critical element of DNA damage response leading to neuronal apoptosis (Kruman et al., 2004). Necdin may prevent mitotic catastrophe of postmitotic neurons by suppressing cell cycle‐promoting proteins such as E2F1 and Cdk1 (see Figure 17). Neurons that aberrantly reenter the cell cycle bear tetraploid nuclei characteristic of *M* (mitosis) phase arrest (Frade & Ovejero‐Benito, 2015; Herrup & Yang, 2007). Since ectopic necdin expression in proliferative cells induces mitotic arrest at the G2/M phase (see Figure 10), necdin may prevent postmitotic neurons from aberrant mitosis and subsequent mitotic catastrophe.

The question then arises as to whether nonplacental animals possess necdin‐like proteins. In Drosophila, MAGE is expressed in neural stem cells (neuroblasts) and postmitotic neurons (Nishimura et al., 2007). Neuronal lineage‐specific knockdown in vivo of Drosophila MAGE increases the population of proliferative neural precursor cells and causes a transient enlargement of the brain (Nishimura et al., 2008). These MAGE‐deficient flies show neuronal apoptosis during development and neurodegeneration at the adult stage. Moreover, MAGE‐mutant flies are hypersensitive to genotoxic agents (Li et al., 2013). These phenotypes of MAGE‐knockdown flies resemble those of Ndn‐null mice, indicating that Drosophila MAGE, like mammalian necdin, exerts antimitotic, anti‐apoptotic and neuroprotective effects. It is conceivable that other single‐type MAGEs, which are expressed preferentially in the nervous systems of nonmammalian vertebrates (Bischof et al., 2003; Lopez‐Sanchez et al., 2007) (see Subsection 4.3), also possess necdin‐like functions. These findings suggest that neurodevelopmental roles of necdin in mammals are inherited from those of ancestral MAGE/NSMCE3.

### 12.2 Implications of neuropathology

PWS, in which neurons completely lack NDN expression, is a complex neurodevelopmental disorder with a variety of neurobehavioral symptoms (Cassidy et al., 2012). Ndn‐null mice show various types of neuronal abnormalities throughout the nervous system at different developmental stages (see Table 1). These mice exhibit physical and behavioral abnormalities such as neonatal respiratory distress, increased spatial learning and memory, increased seizure susceptibilities, central hypothyroidism, increased pain thresholds, abnormal circadian rhythm‐related behaviors, and transient hypotonia (see Subsection 5.3). Some of these features are reminiscent of the PWS symptoms (Cassidy et al., 2012). However, neuronal phenotypes of PWS may be modified from or less obvious than those of Ndn‐null mice. This may be because the absence of NDN expression is compensated, to some extent, by NDN‐homologous MAGEs such as MAGEF1, which is presumedly expressed in PWS in a normal biallelic manner. Furthermore, cortical neurons, which are associated with higher‐order cognitive functions, show abnormalities during development of Ndn‐null mice (see Figures 12, 16, 20, 24). Noteworthily, magnetic resonance neuroimaging studies on the brain morphology of individuals with PWS have revealed low cortical complexities during childhood (Lukoshe et al., 2014) and small gray‐matter volumes in several cortical regions during adulthood (Ogura et al., 2011). These findings raise the possibility that the absence of necdin expression in PWS contributes to cortical structural abnormalities associated with neurobehavioral symptoms.

Necdin expression is altered in mouse models for neurological diseases. In mutant SOD1 (SOD1 G93A)‐over‐expressing mice known as a familial ALS (amyotrophic lateral sclerosis) model, necdin expression is markedly elevated in motor neurons at the presymptomatic stage and down‐regulated at the end stage (Ferraiuolo et al., 2007). This suggests that necdin prevents degeneration of affected motor neurons at the early stage. Fragile X syndrome is caused by the loss of FMRP (fragile X mental retardation protein), whose deficiency affects many brain functions including differentiation of adult neural stem cells. In FMRP‐deficient Fragile X syndrome model mice, necdin expression is up‐regulated in adult neural stem cells (see Figure 3b) that show impaired neuronal differentiation (Liu et al., 2018). SREBP‐1c (sterol regulatory element‐binding protein‐1c) plays a major role in lipid homeostasis in the brain. In SREBP‐1c‐null mice, necdin expression is significantly decreased in hippocampal CA3 region (see Figure 2e) (Ang et al., 2020). These observations suggest that necdin contributes to neuronal phenotypes of these neurological disease model mice.

Mitochondrial abnormalities are implicated in the pathogenesis of neurodegenerative diseases and age‐related neuronal changes (Yankner et al., 2008). Mitochondrial dysfunction generates large amounts of reactive oxygen species, which induce DNA damage leading to neurodegeneration. Necdin stabilizes neuronal PGC‐1α and promotes PGC‐1α‐mediated mitochondrial biogenesis (see Figure 24). Necdin also potentiates the function of Sirt1 (see Figures 16, 23), which facilitates PGC‐1α‐mediated mitochondrial biogenesis (Haigis & Sinclair, 2010). Consequently, necdin most likely prevents oxidative stress‐induced neurodegeneration by promoting the biogenesis and function of neuronal mitochondria.

A multicohort transcriptional meta‐analysis reveals that necdin expression is diminished in major neurodegenerative diseases such as Alzheimer's disease, Parkinson's disease, Huntington's disease and ALS (Li et al., 2014). These data raise the possibility that reduced necdin expression in the brain contributes to the pathogenesis of these human diseases. In Parkinson's disease model mice, viral vector‐mediated NDN over‐expression in vivo in midbrain dopaminergic neurons rescues mitochondrial toxin‐induced neurodegeneration (Hasegawa et al., 2016). Accordingly, viral vector‐mediated NDN transfer into specific neurons at risk will be a promising strategy for the prevention or therapeutic intervention of neurodegenerative diseases. Furthermore, future development of CRISPR‐based genome editing system for transcriptional activation in vivo in postmitotic neurons (Savell et al., 2019) will provide a new avenue to therapeutic application of necdin to neurodevelopmental and neurodegenerative diseases.

### 12.3 Implications of mammalian brain evolution

**FIGURE 30:**
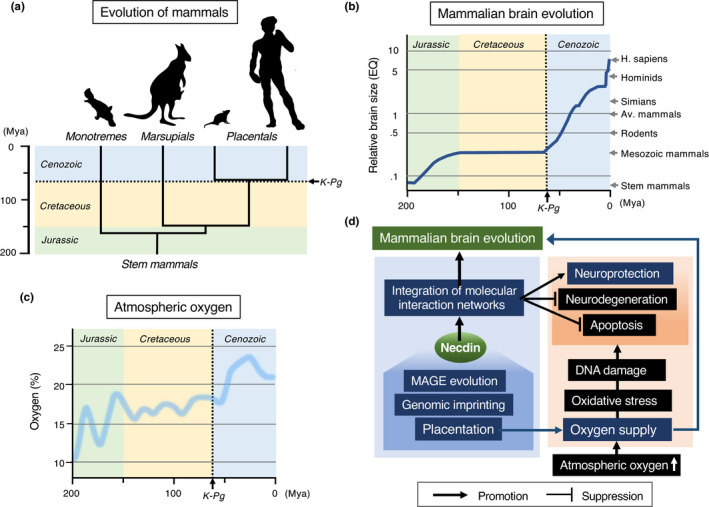
Emergence of necdin is linked to mammalian brain evolution. (a) Evolution of placental mammals. Placental mammals have rapidly diverged in the Cenozoic era after the Cretaceous–Paleogene boundary (K‐Pg) (broken line). Mya, million years ago. Divergence times from Bininda‐Emonds et al. (2007) for monotremes/marsupials and O'Leary et al. (2013) for placentals. (b) Mammalian brain evolution. Relative mammalian brain sizes are represented by encephalization quotient (EQ). EQ values (logarithmic scale) are ~0.25 for Mesozoic mammals, 1 for average (Av.) mammals and >7 for humans (H. sapiens). Adapted from Jerison (1991). (c) Atmospheric oxygen levels in the Mesozoic and Cenozoic eras. Note the doubling of atmospheric oxygen levels over past 200 million years reaching >20% in the Cenozoic era. Adapted from Falkowski et al. (2005). (d) Hypothetical diagram of necdin and mammalian brain evolution. Necdin, which has emerged through the placental mammal‐specific events (genomic imprinting and MAGE evolution), promotes the vitality of mammalian neurons by integrating the protein–protein interaction networks. Necdin may have provided the molecular background behind the brain evolution of placental mammals

Placental mammals (eutherians) have rapidly diverged after the Cretaceous–Paleogene (K‐Pg) boundary associated with the global mass extinction of terrestrial animals including nonavian dinosaurs (O'Leary et al., 2013) (Figure 30a). Furthermore, mammalian brains have evolved rapidly in the Cenozoic era after the K‐Pg boundary (Jerison, 1991) (Figure 30b). The allometric growth of mammalian brain is referred to as encephalization, which correlates with acquisition of intelligence as a result of increased numbers of neocortical neurons. The encephalization quotient (EQ) represents the relative brain size of any animal species in which brain and body sizes are known (Jerison, 1970). Humans (*Homo sapiens*) have the highest EQ (>7) among all existent and preexistent animal species, whereas EQs of Mesozoic mammals are ~0.25. This indicates that the mammalian brain size rapidly increased during the Cenozoic era. However, the mechanism behind the rapid mammalian brain evolution has been a riddle of evolutionary biology.

Human brain contains approximately 86 billion neurons (Herculano‐Houzel, 2009). The human brain, for example, weighs only ~2% of the total body mass but accounts for ~20% of total resting metabolism (Attwell & Laughlin, 2001). Accordingly, the brain requires large amounts of oxygen indispensable for efficient ATP production in mitochondria (Bailey, 2019). Since most of mammalian neurons arise during the embryonic period, the mammalian brain evolution may have been achieved only if oxygen was sufficiently supplied to embryonic neurons. Evolution of the placenta enabled embryonic neurons to take in large amounts of oxygen efficiently for maintaining their activities. Furthermore, the placentation was accompanied by the two genetic events: Genomic imprinting has contributed to the allometric growth of mammalian brain in a parent‐of‐origin‐specific manner (Keverne, 2013), and the MAGE evolution has created necdin and its related MAGEs that promote neuronal vitality.

Atmospheric oxygen levels approximately doubled from ~10% to ~21% over the past 200 million years (Falkowski et al., 2005) (Figure 30c). Therefore, it is presumed that high atmospheric oxygen levels in the Cenozoic era have been advantageous to huge numbers of brain neurons in placental mammals. However, high oxygen levels adversely generate considerable amounts of cytotoxic reactive oxygen species (Bailey, 2019), which cause DNA damage leading to neuronal apoptosis and degeneration (Klein & Ackerman, 2003). Through the placentation‐associated genetic events, necdin has emerged to promote the vitality of postmitotic neurons in mammals (Figure 30d). Consequently, necdin may have paved the way for the mammalian brain evolution reaching the human brain.
